# Ibuprofen supports macrophage differentiation, T cell recruitment, and tumor suppression in a model of postpartum breast cancer

**DOI:** 10.1186/s40425-018-0406-y

**Published:** 2018-10-01

**Authors:** Nathan D. Pennock, Holly A. Martinson, Qiuchen Guo, Courtney B. Betts, Sonali Jindal, Takahiro Tsujikawa, Lisa M. Coussens, Virginia F. Borges, Pepper Schedin

**Affiliations:** 1 Department of Cell, Developmental and Cancer Biology, Oregon Health & Science University, 2720 SW Moody Ave, Portland, OR 97201 USA; 2 WWAMI School of Medical Education, University of Alaska Anchorage, 3211 Providence Dr, Anchorage, AK 99508 USA; 3 Department of Otolaryngology-Head and Neck Surgery, Kyoto Prefectural University of Medicine, Kyoto City, Kyoto Prefecture Japan; 4 Department of Medicine, Division of Medical Oncology, University of Colorado Anschutz Medical Campus, MS8117, RC-1S, 8401K, 12801 E 17th Ave, Aurora, CO 80045 USA; 5 Young Women’s Breast Cancer Translational Program, University of Colorado Cancer Center, University of Colorado Anschutz Medical Campus, 1665 Aurora Court, Aurora, CO 80045 USA; 6 Knight Cancer Institute, Oregon Health & Science University, 2720 SW Moody Ave, Portland, OR 97201 USA

**Keywords:** Macrophages, T cells, Postpartum breast cancer, Multiplex IHC, Ibuprofen, Tumor microenvironment, NSAIDs

## Abstract

**Background:**

Women diagnosed with breast cancer within 5 years postpartum (PPBC) have poorer prognosis than age matched nulliparous women, even after controlling for clinical variables known to impact disease outcomes. Through rodent modeling, the poor prognosis of PPBC has been attributed to physiologic mammary gland involution, which shapes a tumor promotional microenvironment through induction of wound-healing-like programs including myeloid cell recruitment. Previous studies utilizing immune compromised mice have shown that blocking prostaglandin synthesis reduces PPBC tumor progression in a tumor cell extrinsic manner. Given the reported roles of prostaglandins in myeloid and T cell biology, and the established importance of these immune cell populations in dictating tumor growth, we investigate the impact of involution on shaping the tumor immune milieu and its mitigation by ibuprofen in immune competent hosts.

**Methods:**

In a syngeneic (D2A1) orthotopic Balb/c mouse model of PPBC, we characterized the impact of mammary gland involution and ibuprofen treatment on the immune milieu in tumors and draining lymph nodes utilizing flow cytometry, multiplex IHC, lipid mass spectroscopy and cytokine arrays. To further investigate the impact of ibuprofen on programming myeloid cell populations, we performed RNA-Seq on in vivo derived mammary myeloid cells from ibuprofen treated and untreated involution group mice. Further, we examined direct effects of ibuprofen through in vitro bone marrow derived myeloid cell cultures.

**Results:**

Tumors implanted into the mammary involution microenvironment grow more rapidly and display a distinct immune milieu compared to tumors implanted into glands of nulliparous mice. This milieu is characterized by increased presence of immature monocytes and reduced numbers of T cells and is reversed upon ibuprofen treatment. Further, ibuprofen treatment enhances Th1 associated cytokines as well as promotes tumor border accumulation of T cells. Safety studies demonstrate ibuprofen does not impede gland involution, impact subsequent reproductive success, nor promote auto-reactivity as detected through auto-antibody and naïve T cell priming assays.

**Conclusions:**

Ibuprofen administration during the tumor promotional microenvironment of the involuting mammary gland reduces overall tumor growth and enhances anti-tumor immune characteristics while avoiding adverse autoimmune reactions. In sum, these studies implicate beneficial prophylactic use of ibuprofen during the pro-tumorigenic window of mammary gland involution.

**Electronic supplementary material:**

The online version of this article (10.1186/s40425-018-0406-y) contains supplementary material, which is available to authorized users.

## Background

Young women diagnosed with breast cancer within 5 years of a pregnancy experience increased rates of metastasis and lower overall survival than age-matched nulliparous women, even after controlling for patient age, tumor biological subtype, clinical stage, and year of diagnosis [1]. To better understand the poor prognosis associated with this reproductive window, researchers have considered whether it is the biology of pregnancy or the postpartum period that is responsible for increased mortality. Importantly, only a diagnosis in the postpartum period has been identified as an independent risk factor for poor outcomes [2]. Consequently, cases diagnosed within the 5 year postpartum period have been specifically defined as postpartum breast cancer (PPBC) [3]. The mechanism underlying postpartum promotion of breast cancer progression during this window of susceptibility is an active area of research.

Using rodent models, we and others have observed that the transient event of weaning-induced mammary gland involution facilitates mammary tumor promotion [4–6]. Mammary gland involution is a developmentally regulated process whereby 80–90% of the secretory mammary epithelium undergoes programmed cell death. This serves to return the lactating gland to a non-secretory state. Utilizing xenograft mouse models of PPBC, we have reported enhanced tumor growth and dissemination when tumor cells are implanted into the involuting mammary gland compared to age-matched nulliparous hosts [7]. Furthermore, in human tissues and mouse models, we and others have identified biological processes of mammary gland involution including increased lympangiogenesis [8], fibroblast activation [9], extracellular matrix (ECM) deposition [10, 11], and the presence of wound-healing-like myeloid cell polarization [12]. All of these processes are documented to contribute to primary or metastatic tumor progression. In sum, these findings support a role for the involuting mammary gland microenvironment in governing tumor outcomes in PPBC patients [13]. At the same time, these studies identify involution as a transient developmental window that can be targeted for the prevention and treatment of life-threatening breast cancer [14]. A unique treatment opportunity lies in the ability to limit duration of intervention to the “window of risk”. Such a directed approach is anticipated to reduce complications associated with current long-duration chemoprevention strategies and may enhance efficacy of treatment as well.

Since the involution environment of the mammary gland has many distinct tumor promotional processes, identifying a single interventional strategy is challenging. Nonetheless, in rodents, we have identified the prostaglandin generating enzyme cyclooxygenase-2 (COX-2) as an influential player for many of the pro-tumorigenic processes that occur during involution. COX-2 protein and associated prostaglandin production are elevated in the involution microenvironment and in postpartum tumors compared to tumors developing in the nulliparous host. Further, non-steroidal anti-inflammatory drugs (NSAIDs) have been identified as a possible way of targeting this pro-tumorigenic COX-2 activity. When administered, NSAIDs have been shown to blunt mammary lymphangiogenesis [8], alter ECM deposition [15–17], and reduce fibroblast activation [9], as well as lower overall tumor growth and dispersion [7]. Conceptually, NSAIDs are thought of as immune-modulatory agents; therefore, an understanding of the immune microenvironment of involution is necessary to fully characterize the mechanisms by which NSAIDs impair tumor promotion.

It is known that mammary gland involution demonstrates “inflammation” as defined by dramatically increased infiltrate of numerous immune cell subtypes [6, 18], including recently documented recruitment of Th17 T cells [19]. The normal involuting mammary gland also possesses strong regulatory and wound-healing-like immunity including the increased presence of suppressive myeloid cell subsets, FoxP3+ T cells, and elevated levels of TGFbeta, IL-10, IL-4 and IL-13 [12, 18, 19]. Furthermore, phagocytosis of apoptotic cells, including mammary epithelial cells [20], induces active immune suppression [5, 21, 22] that may contribute to the observed impairment of antigen-specific activation of T cells that takes place during involution [19]. Based on these observations, it is quickly surmised that under such immunological influences anti-tumor immunity would be impaired. Impaired immunity may significantly contribute to the tumor promotional attributes of the involuting gland. What is less immediately apparent is how NSAID administration might impact these immunological features to enhance anti-tumor immunity.

Investigating how NSAIDs’ anti-inflammatory activity interfaces with the immune milieu of the involuting gland remains an essential avenue for advancing NSAIDs as preventive and therapeutic agents in PPBC [14]. What is known of NSAID use in response to injury or in pathologic conditions such as arthritis and multiple sclerosis is that they exert “anti-inflammatory” effects through inhibition of prostaglandin E2 (PGE2) production. PGE2 has been identified as one of the key agents in driving tissue swelling [23], analgesia/nociception pathways [24], and immune cell recruitment in response to tissue damage [25]. The mechanism of PGE2 mediated tissue inflammation has been reported to involve promotion of IL-6 [26] production and enhancement of polymorphonuclear (PMN) cell degranulation [27]. Together these processes drive an inflammatory milieu that supports increased differentiation of pathogenic Th17 T cells [28, 29]. Thus, one mechanism by which NSAIDs exert anti-inflammatory activity is through suppression of PGE2 and downstream activation of the IL-6/PMN/Th-17 cascade. In contrast, numerous other studies bring to light PGE2’s promotion of regulatory/tolerogenic immunity. These include demonstrated and direct roles in promoting regulatory T cell differentiation [30, 31], programming tolerogenic dendritic cells [32, 33], decreasing sensitivity of T cells to IL-2 [34], inhibition of monocyte differentiation [35], and persistence of myeloid derived suppressor cells (MDSCs) [36–38]. Interestingly in all of these scenarios, inhibition of these processes by administration of an NSAID would be predicted to enhance anti-tumor immune responses.

In the following studies, we sought for the first time to understand the impact of NSAIDs on tumor myeloid and T cell immune milieus in an immune competent murine model of PPBC. Utilizing the D2A1 mammary tumor cell line and the syngeneic Balb/c mouse host, we demonstrate with flow cytometry and multiplex immunohistochemistry (IHC) that tumors within the microenvironment of the involuting mammary gland have distinct immunological features from those of nulliparous hosts. For instance, involution group tumors display increased presence of immature myeloid cells, decreased presence of mature macrophages and reduced numbers of intratumoral CD4 T cells. Treatment with ibuprofen beginning at time of weaning, significantly reduced involution associated tumor burden, increased tumor border T cells, and increased both the number of mature macrophages as well as the intratumoral cytokines associated with the promotion of Th1 and cytotoxic anti-tumor immunity (TNFa, IL-12 and IL-2). These observations are reinforced by ibuprofen treatment of involution without tumors both in vivo and in vitro*,* which demonstrate macrophage and monocyte maturation as targets of ibuprofen treatment.

In first steps to consider ibuprofen as a chemopreventive strategy in young women’s breast cancer, we employed a mouse model to investigate whether there may be unintended consequences to elevated Th-1 related immunity instigated by low dose of ibuprofen. Through a mouse multi-parity study, we find no impact of ibuprofen on reproductive success, offspring growth, glandular morphology, nor detect any elevation in autoantibody production. Furthermore, we report that in the absence of tumor, ibuprofen does not reverse the physiologically normal inhibition of antigen-specific naïve T cell activation that occurs during normal mammary gland involution. Thus we propose NSAIDs as a potential safe agent for promoting anti-tumor immunity during the postpartum window of tumor promotion.

## Methods

### Preclinical mouse model

University of Colorado and Oregon Health & Science University IACUC committees approved all mouse procedures in compliance with NIH guidelines and accepted AALAC recommendations. For specific reproductive stages, Balb/c female mice (Jackson Laboratories) were bred with male C57Bl/6 male mice (Jackson Laboratories) in ventilated micro-isolator cages, with 12 h light/dark cycles. Breeding of the two allogeneic strains is done to maximally induce fetal-maternal tolerance mechanisms, modeling those present in all humans. Such tolerance is known to shape maternal immunity during pregnancy and could influence immunity in subsequent developmental stages. In all studies, all nulliparous and involution cohorts are vendor provided Balb/c female mice. Two days post-parturition litter sizes were normalized (6–8 pups). Weaning was initiated at lactation days 10–14 (denoted LACT). For mice receiving tumor, mice were anesthetized with isoflurane (VetOne) and injected with 20,000 D2A1 cells in 20 μl PBS into the right and left 4th mammary gland fat pads of involution day 1 and age-matched, cage mate, nulliparous group mice. The D2A1 cell line was a generous gift from Dr. Ann Chambers (Ontario, Canada) and was maintained as previously described [39]. Ibuprofen at the indicated dose was added to pulverized standard chow and mixed dry, then mixed with water, made into chow pellets and dried thoroughly. Ibuprofen chow was given ad libitum where indicated. For involution mice, ibuprofen chow was given on the day pups were removed (Involution day 1) and for the duration of the tumor study. Tumor burden as volume was evaluated by caliper measurement twice weekly and calculated as width x length x length × 0.5. Mice were sacrificed approximately 3 weeks post-injection for tissue and blood isolation. Lymph node-free mammary glands 4 and 5 and tumor tissues were collected for flow cytometry, immunohistochemistry and western blot analyses.

### Multiparity mouse model

Female Balb/c mice were bred as described above with C57Bl/6 J male mice. Two days post-parturition litter sizes were standardized to 6–7 pups and weaning initiated on day 10 of lactation. Control mice were given standard mouse chow and ibuprofen treated mice were given standard mouse chow supplemented with 300 mg/kg ibuprofen starting on day one of involution and ending on day 14 of involution. This breeding process was repeated twice and multiparous mice were sacrificed at 5 weeks post-involution for tissue and blood isolation.

### Tumor digestion and immune cell flow cytometry

Single cell suspensions were prepared from mammary gland or tumor dissection by dicing tissue into 1 mm fragments, followed by digestion for 1 h at 37 °C in 50 μg/mL Liberase (Roche) in Hanks Balance Salt Solution or with Collagenases II and IV (2.5 mg ml-1 Worthington) and DNase I (0.5–2.5 mg ml-1 Worthington) in HBSS with calcium and magnesium for 30 min with agitation at 37C by rotation. Digestion mixtures were quenched using RPMI containing 10% FBS and filtered through a 70 μm nylon strainer (BD Biosciences). Filtered cell digest of mammary gland was stained with trypan blue and viable cell count determined by hemocytometer analysis. Single cell suspensions were incubated for 20 min at 4 °C with 1 ul of the following fluorophore-conjugated anti-mouse antibodies in a final volume of 100 ul of FACS buffer (CD11b-APC-eFluor780 (M1/70), CD3-APC-eFluor780 (17A2), CD4-FITC (RM4–5), CD8-PerCP-eFluor710 (eBioH35–17.2) (all from eBioscience) and/or, Ly6G/C (GR1) -PE (RB6-8C5, BD), CD45-Pacific Blue (30-F11, Invitrogen), and F4/80-FITC (Cl:A3–1, AbD Serotec)) or CD45-PE-Cy7 (30-F11, Thermofisher), MHCII-eFluor-450 (M5/114.15.2, Thermofisher), B220-BV785 (RA3-6B2, Biolegend), CD11b-BV711 (M1/70, Biolegend), CD11c-BV605 (N418, Biolegend), F4/80-APC (BM8, Thermofisher), Ly6C-PerCP-Cy5.5 (HK1.4, Thermofisher), Ly6G-APC-Cy7 (1A8, Thermofisher), CD103-FITC (2E7, Thermofisher), CD70-PE (FR70, Thermofisher), CD80-BV650 (16-10A1, Biolegend), CD86-AF700 (GL-1, Thermofisher) and Live Dead Aqua (Invitrogen) using either predetermined titrations or the manufacture’s recommended concentrations. For co-staining of intracellular and membrane-expressed antigens, cells were labeled first with the membrane-targeted antibodies, then fixed and permeabilized using the Foxp3 Fixation/Permeabilzation kit (eBioscience) according to manufacturer’s protocol. Cells were then stained for intracellular targets or isotype control (eBioscience). Data acquisition was performed using either a Gallios (Beckman Coulter) or Fortessa (Becton Dickinson) flow cytometer and either Kaluza (Beckman Coulter) or FlowJo (Treestar) software was used for analysis.

### Mammary gland and tumor tissue preparation for RNA, whole protein and lipid-mass spec analysis

Flash frozen #4 mouse mammary gland with lymph node removed or tumor tissue was pulverized in liquid nitrogen using a mortar and pestle and aliquoted for subsequent, lipid, protein and RNA assays. Thirty milligram of pulverized mammary gland were shipped on dry ice for absolute lipid quantification analysis by the Mass Spectrometry Lipidomics Core Facility, University of Colorado Anschutz Medical Campus, Aurora, Colorado.

### Immunoblot

Flash frozen aliquots of tissue were prepared into lysates and total protein concentration determined for use in western blots as described [40]. Mammary lysates were loaded by equal protein, run under reducing conditions and transferred to PVDF membranes as previously reported [4]. Membranes were incubated (overnight at 4 °C unless stated otherwise, diluted in 5% BSA in TBS-T) with the following antibodies: mouse polyclonal anti-COX-2 (1:1000, Cayman Cat # 160106); and rabbit polyclonal anti-GAPDH (1:1000; Sigma Cat# G9545) for 1 h at room temperature, followed by matched secondary antibody detection utilizing HRP mediated chemiluminescence and x-ray film detection.

### Bone marrow isolation, culture and MTT assay

Bone marrow was isolated from tibia and femurs of 8 to 12 week old nulliparous female Balb/cJ mice and filtered through a 100 um filter. Bone marrow cells were incubated with Red Blood Cell Lysis buffer (eBioscience) for 1 min and then washed with PBS. Cells were plated at 2–3 × 10^5^ cells/mL in 24 well plates in bone marrow medium (RPMI supplemented with 3% FCS, 1% antibiotic-antimycotic, 1% gentamicin Life Technologies), including granulocyte-macrophage colony stimulating factor (GM-CSF; 20 ng/mL) and IL-4 (10 ng/mL) (Peprotech) at 37 °C in 5% CO_2_ for 5 days. PGE_2_ (2.6 μmol/L) and/or ibuprofen (25, 50, 75, 100 μmol/L) was added to some wells at the beginning of the 5-day culture. Half of the medium was replaced every 48 h with bone marrow medium. Cells were isolated after 5 days, stained, and analyzed by flow cytometry for expression of CD11b and F4/80. To evaluate ibuprofen's impact on cell viability, wells were stained by MTT assay 24 and 48 h after introduction of ibuprofen. For this 10 μl of 3 mg/mL MTT, (3-(4,5-dimethylthiazol-2-yl)-2,5-diphenyl tetrazolium bromide – Sigma Millipore, Cat # CT-02) in PBS pH 7.4 was added per 100 μl of media covering cells on tissue culture plates and incubated for 4 h at 37^o^ C and 5% CO2. An equal volume of isopropanol with 0.04 N HCl solution was added to each well and mixed thoroughly. Absorbance at 570 nm with a 630 nm reference wave length was recorded for every well. Data were normalized to wells without ibuprofen as 100% viability.

### RNA isolation, cDNA synthesis, and quantitative RT-PCR

RNA was isolated from 30 mg of flash frozen mammary tumor tissue using 1 mL TRIZOL reagent (Life Technologies) following the manufacture instructions. RNA was resuspended in 50 μl of nuclease-free water and RNA concentration and purity determined by 260 nm and 280 nm fluorescence using the Nanodrop 2000 UV-Vis spectrophotometer (ThermoScientific). cDNA was synthesized from 1 μg total RNA using the iScript cDNA Synthesis kit (Bio-Rad). For quantitative RT-PCR, 1 μl of cDNA was amplified with SYBR green (Bio-Rad) using forward and reverse primers at a concentration of 1 μM each, and brought up to a final reaction volume of 20 μl with nuclease-free water. The following mouse primers were used from Integrated DNA Technologies: *Gzmb*, forward: 5′- CAG GTG GCA GGA TCT GAA TAA A-3′, reverse: 5′- GGC AGA AGA GGT GTT CCA TT-3′; *IL1B*, forward: 5′-TGG AGA GTG TGG ATC CCA AGA AAT-3′, reverse: 5’-TGC TTG TGA GGT GCT GAT GTA CCA-3′; *IL10*, forward: 5’-GCT CTT GCA CTA CCA AAG CCA CAA-3′, reverse: 5′-AGT AAG AGC AGG CAG CAT AGC AGT-3′; *IL12*, forward: 5′-TGA TGA TGA CCC TGT GCC TTG GTA-3′, reverse: 5′-ATT CTG AAG TGC TGC GTT GAT GGC-3′; *TNFA*, forward: 5’-TCT CAT GCA CCA CCA TCA AGG ACT-3′, reverse: 5’-ACC ACT CTC CCT TTG CAG AAC TCA-3′; and *Actb*, forward: 5’-GCA ACG AGC GGT TCC G-3′; reverse: 5’-CCC AAG AAG GAA GGC TGG A-3′. Real-time RT-PCR conditions were: 3 min, 95 °C; 10 s, 95 °C, 1 min 60 °C (42 cycles); 1 min, 95 °C, 1 min 55 °C. Primer specificity was confirmed via melt-curve analysis. Data were collected using the MyiQ Single Color Real-Time PCR Detection System with iQ5 software (Bio-Rad), and gene expressions were normalized to actin using the delta-delta Ct method.

### Multiplex IHC

Formalin fixed, paraffin embedded tissues were sectioned at 4 μm. On day of staining, paraffin was melted from slides by 2 h bake in 60 °C oven and then slides rehydrated through sequential emersion in xylene and gradient alcohols into water and finally DAKO – Target Retrieval Solution (TRS-pH 6). Tissues were retrieved in Dako-TRS by pressure cooker at 115 °C for 20 min and then cyclically probed as described previously [41] in the following order with the indicated antibody, dilution and incubation times. Cycle 1 GrB (Abcam, Ab4059, 1:300, 1Hr), Cycle 2 CD8a (eBioscience, 14–0195(4SM16), 1:100, 2Hr), Cycle 3 CD3 (Abcam, ab16669, 1:100, 1Hr), Cycle 4 CD45 (BD Pharmingen, 550539 (Clone 30F11), 1:50, 2Hr), Cycle 5 Ki67 (Neomarkers, RM-9106-s, 1:100, 1Hr), Cycle 6 Rorgt (eBioscience-Thermofisher, 14–6988-80, 1:75, 2Hr), Cycle 7 Foxp3 (eBioscience, 14–5773-82, 1:100, 2 Hr). Detection of primary antibodies was performed with either anti-rabbit or anti-rat horseradish peroxidase polymers (anti-rabbit or anti-rat Simple Stain MAX PO Histofine Peroxidase Polymer, Nichirei Biochemicals) followed by detection with peroxidase substrate 3-amino-9-ethylcarbazole (AEC), then digital scanning was performed using Aperio ImageScope AT2 (Leica Biosystems) at 20× magnification. Image processing of muliplex IHC images including coregistration, AEC chromagen and cellular deconvolution were performed as previously reported [41] utilizing CellProfiler (Broad Institute). Image cytometry was performed utilizing FCS-Express software (DeNovo Software, Glendale, CA). Due to lack of robust antibody availability for CD4, CD3 + CD8- cells were considered as CD4+ T cells for these analyses.

### Cytokine array

Cytokine profiling was performed using Meso Scale Discovery (MSD) Multi-spot plates and an MSD Sector Imager 2400 reader (Meso Scale Discovery). Using the MSD 10-Plex Mouse Proinflammatory Panel I, a total of 10 cytokines were measured simultaneously (IFN-γ, IL-1β, IL-2, IL-4, IL-5, IL-6, IL-10, IL-12p70, KC/GRO, and TNF-α) according to manufacturer’s instructions. Briefly, tumor and mammary gland lysates were diluted 1:10 in supplied assay diluent. Sample lysate, 50 μl, was added in duplicate to the appropriate wells in a 96 well plate. The array was incubated for 3 h at room temperature, while shaking. The array was then washed with phosphate-buffered saline plus 0.05% Tween 20, and 25 μL of detection antibody reagent was added and incubated for 2 h while shaking. The array was washed, detection buffer added and results read using an MSD Sector Imager 2400 incorporating a charge-coupled device. Sample cytokine concentrations were determined with Discovery Workbench software (Meso Scale Discovery).

### Measurement of auto-antibodies

The presence of anti-double stranded DNA (anti-dsDNA) antibodies in serum from multiparous mice was determined using a mouse ELISA kit (5110, Alpha Diagnostic), following manufacture instructions and diluting mouse serum at 1:100.

### Macrophage – Monocyte RNASeq – Gene set enrichment and pathway analysis

Mammary gland tissues were digested and processed into single suspension as described previously. Cells were then flow sorted based upon levels of F4/80 expression and lineage negative marker. F4/80+ cells were confirmed to be CD45+ based upon flow cytometry (Additional File 2, Figure S2a). RNA was isolated as described above and library preparation was performed by the Massively Parallel Sequencing Shared Resource at Oregon Health & Science University, using a TruSeq RNA Sample Preparation V2 kit. Four samples were pooled per lane and sequenced on an Illumina HiSeq 2500 with 100-bp single-ended reads. The resulting data were converted to FASTQ format using Bcl2Fastq software (Illumina). Reads were aligned to the mouse reference genome, build GRCm38, using STAR 2.4.2a. STAR performed counting of reads per gene as defined in Ensembl build 81 (GENCODE version m6). Read counts were adjusted to a common non-zero minimum via addition of uniform minor background counts. Read count distributions were normalized across samples using cyclic Loess normalization by R software. Normalized read counts were log2-transformed for further analysis. Z-scores plots of differential expressed genes (determined by plots of fold change and *p*-value z-score,) were generated by inputting absolute counts for designated genes into the Morpheus web tool (https://software.broadinstitute.org/morpheus) and selecting for z-score transformation of data. Pathway analysis was performed through either Panther database (http://pantherdb.org/) with pre-designated DE genes and DE gene values or with Gene Set Enrichment Analysis Software (GSEA - http://software.broadinstitute.org/gsea) on the complete gene expression profile for each sample. PCA plots were constructed from complete gene expression data using the shiny STARTApp (https://github.com/jminnier/STARTapp).

### TCGA – Breast Cancer Kaplan-Meier analysis

Key genes and transcription factors determined from differential expression analysis of macrophages, monocytes, and tumors with and without ibuprofen in the above murine studies were selected as genes of interest for determination of gene expression correlation with survival outcome in humans. Kaplan-Meier plots were produced with the UCSC-Xena Functional Genomics Browser tool and the TCGA-Breast Cancer data sets with numbers of samples indicated in brackets for gene expression Hi (red) and Lo (blue or gray) and *p*-values displayed determined by the software (https://xenabrowser.net/heatmap/).

### In vivo T cell activation assay

T cell receptor transgenic mice recognizing a CD4 epitope in chicken ovalbumin (Do11.10) on the Balb/c background were obtained from Jackson Labs (Stock #003303, C.CG-Tg(Do11.10)10Dlo/J) and bred to maintain a colony in-house. For each day of transgenic T cell transfer, spleens from two naïve female Do11.10 mice were homogenized to single-cell suspension, pooled and red blood cells lysed by ACK buffer. CD4+ T cells enrichment was performed by antibody mediated magnetic negative selection (Miltenyi Biotec-130-104-453). After isolation, an aliquot of cells was stained to determine purity and levels of transgenic TCR expression (Ebioscience/Thermofisher antibody clone KJ1–26, Cat# 17–5808-80) with greater than 90% CD4+ T cells enrichment observed across study days. Cellularity was adjusted to deliver 150,000 naïve Balb/c transgenic T cells through tail vein injection into vendor provided Balb/c mice two days prior to antigen delivery in either involution or nulliparous hosts, with or without 300 mg ibuprofen/ Kg of chow. Two days later whole chicken ovalbumin (Worthington Biochemical Corp, Cat# LS003059, 10 μg in 10 μl 1xPBS) was injected into either the left or right #4 mammary fat pad, and the opposite mammary fat pad received equal volume PBS. Five days later inguinal lymph nodes were harvested, minced and digested to single cell suspension with collagenases II and IV (2.5 mg ml-1 Worthington) and DNase I (0.5–2.5 mg ml-1 Worthington) in HBSS with calcium and magnesium for 30 min with agitation at 37C. Digests were filtered (100 μm) and red blood cells lysed (eBioscience) per manufacturer’s instructions and stained with extracellular antibodies (CD45, 30-F11; CD4, RM4–5; D011.10 TCR, KJ1–26). On day of flow, a known quantity of absolute counting beads were added to samples (C36950 Invitrogen) and flow cytometry performed for absolute quantification of transgenic T cells. To assess T cell activation and account for mouse-to-mouse variation in T cell seeding, the absolute number of transgenic T cells in the antigen experienced gland was divided by the number of T cells in the contralateral PBS injected gland.

### Statistical analysis and hierarchical clustering

Determination of statistically significant differences between two groups were based upon two-tailed Student’s t-test between the indicated groups with *p* < 0.05 (*),  *p* < 0.01 (**), *p* < .001 (***) unless otherwise stated in the figure legend. Statistical analyses were performed using GraphPad Prism v.5 software. All data are expressed as mean +/− standard error of the mean (SEM) unless otherwise noted. Hierarchical clustering of multiplex IHC data was performed with the Morpheus web-based tool (https://software.broadinstitute.org/morpheus) utilizing average linkage methodology for Euclidean distances and excluding final tumor volume as a clustering parameter. Colors shown within a given column are based upon relative max and min values derived and represent the values of the indicated parameter.

## Results

### Mammary gland involution imprints lasting tumor immunity

**Fig. 1:**
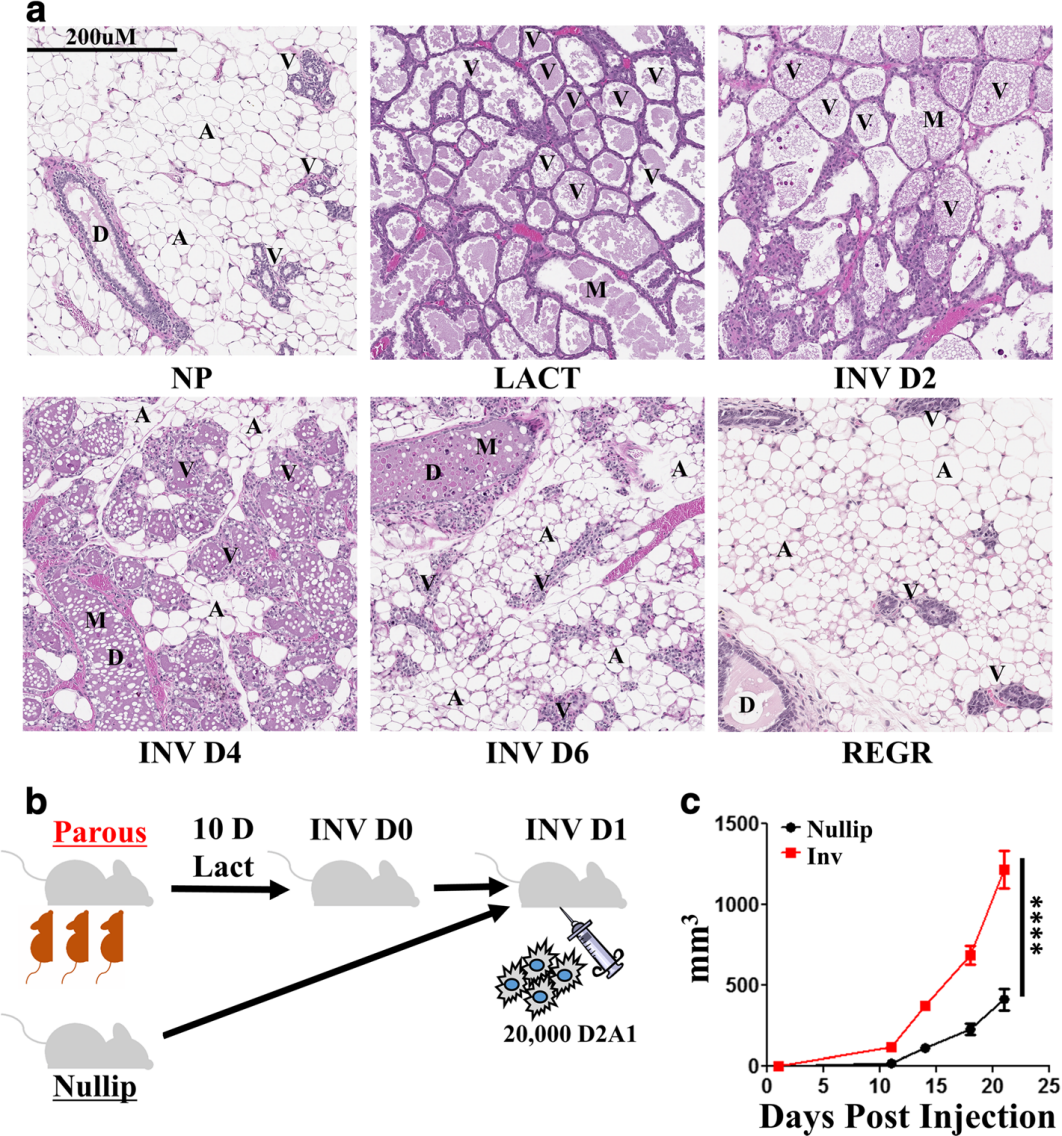
Weaning-induced mammary gland involution is a dynamic, tissue remodeling process that promotes breast cancer. **a** H&E sections of Balb/c mouse mammary glands across a reproductive cycle show the presence of ductal (D) and alveolar (V) epithelial structures, adipocytes (A), and milk (M) secretions within luminal spaces from nulliparous (NP), lactation (LACT), involution day two, four and six (INV D2, INV D4, and INV D6) and 6 weeks regressed (REGR) glands. **b** Groups of age matched Balb/c mice were bred (parous/involution, *n* = 8) or not (nulliparous, *n* = 6). Involution cohorts lactated for 10 days and pups removed to initiate synchronous mammary gland involution. One day post-weaning, 20,000 D2A1 syngeneic breast cancer line tumor cells were implanted orthotopically in the 4th mammary gland of involution or nulliparous Balb/c mice. **c** Tumor growth was monitored for 3 weeks before mice were euthanized for tissue collection and analyses. Determination of statistical differences between NP and INV were based upon two-tailed Student’s t-test comparison with *p* < 0.0001 (****)

The cellular composition of the adult mammary gland is highly dynamic and dependent on reproductive state. In adult nulliparous mice (Fig. 1a), the gland is predominantly composed of adipocytes (A) and ductal epithelium (D) with sparse alveoli (V) (Fig. 1a, NP). Starting in pregnancy and reaching its zenith in lactation, the milk (M) secreting alveolar epithelial content of the gland increases dramatically (Fig. 1a, LACT). At parturition, in the absence of nursing, or upon cessation of lactation, 80–90% of the alveolar epithelium undergoes programmed cell death. Epithelial cell loss occurs concurrent with adipocyte repopulation, in a process known as mammary gland involution. Within 48 h of the onset of involution, shed and dying cells can be seen within the alveolar and ductal lumen (Fig. 1a, InvD2). Although large in scale, involution in the rodent model is rapid with the majority of epithelium eliminated by 6 days post-weaning (Fig. 1a, InvD6). Upon complete regression (Fig. 1a, REGR) the histologic features of the gland resemble the non-secretory, nulliparous state.

Apart from these dramatic tissue morphological changes, involution is also characterized by a dynamic local recruitment of diverse immune cells. Previous gene expression analyses found the early days of involution to resemble an acute phase inflammatory response [42, 43] that subsides and is replaced by robust wound-healing-like immune signatures (IL-4, IL-10, TGFbeta, IL-3) [5, 12, 18, 21]. Conceptually, these gene expression studies are consistent with a functional role for immune cells in facilitating tissue remodeling during the dynamic window of weaning-induced involution.

In the present work, we address whether the transitory local immune response of the involution window has a lasting impact on mammary tumors and related adaptive immunity. To ensure full engagement of fetal-maternal tolerance mechanisms that might shape subsequent mammary gland immunity, we bred immune competent female Balb/c mice to C57BL6/ males. The Balb/c females had normal progression of pregnancy and litter sizes, indicative of fetal-maternal tolerance. After birth, pup number was normalized between dams, followed by 10 days of nursing, at which point pups were removed from the dams to initiate synchronized mammary gland involution. One day post-weaning, 20,000 syngeneic D2A1 Balb/c mammary tumor cells were orthotopically implanted into the inguinal (4th) mammary fat pads of Balb/c involution mice and age matched, nulliparous female controls (Fig. 1b). In this model, a significant and persistent increase in tumor size and tumor growth trajectory is observed in the involution hosts for at least 21 days (study end) compared to nulliparous hosts (Fig. 1c), even though involution is almost completely resolved by 7 days post tumor seeding. These observations illustrate that in the presence of an intact immune system, tumor cells that experience the involuting microenvironment have a growth advantage which is maintained even after the gland returns to a nulliparous-like state.

**Fig. 2:**
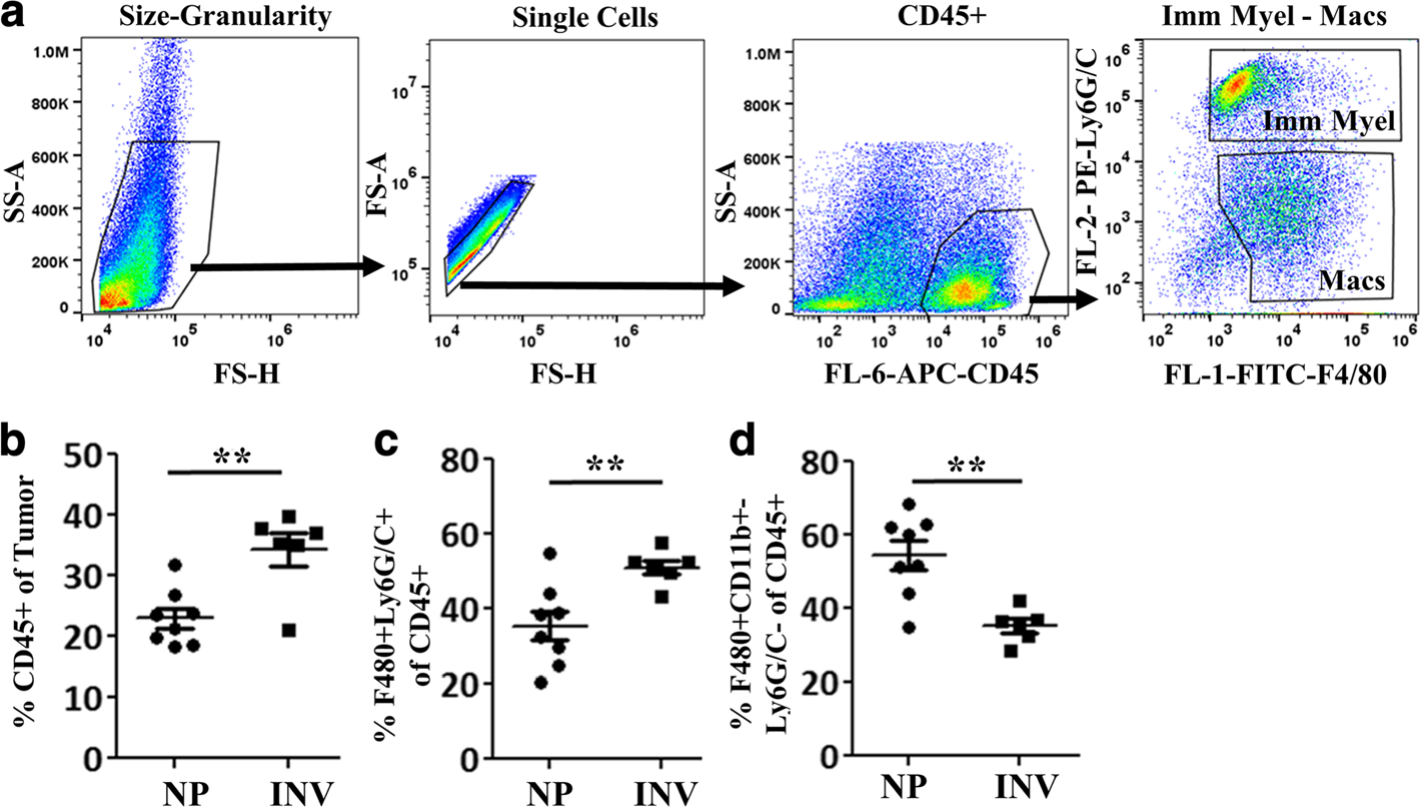
Involution group tumors are enriched for immature myeloid cells. After three weeks of in vivo growth, tumors from nulliparous (NP) and involution (INV) groups were resected, digested into single cell suspensions, and myeloid populations quantified by flow cytometry. **a** Representative gating schema based upon scatter, single cells, CD45+, Ly6G/C and F4/80. INV compared to NP group tumors have **b** increased CD45+ infiltrate, **c** elevated immature myeloid cell/monocyte population as classified as Ly6G/C+ F4/80+ cells, and **d** decreased mature macrophages classified as the F4/80+ Ly6G/C– population. Determination of statistical differences between NP and INV were based upon two-tailed Student’s t-test comparison with *p* < 0.01 (**)

To address whether involution’s pro-tumorigenic influence on the tumor immune milieu extends past the involution window, we assessed tumor immune cell composition at 3 weeks post tumor cell injection. We previously reported that during the later stages of involution, in the absence of tumor, there exists a transient wound-healing-like immune microenvironment with increased “M2-like” myeloid cells [12, 18]. Knowing this, we assessed how involution differentially impacts subsets of the myeloid cell compartment within the tumors. To this end, flow cytometric analysis of tumor single cell digests was performed to examine the relative contribution of mature macrophages (CD45 + F4/80 + Ly6G/C-) and immature monocytes (CD45 + F4/80 + Ly6G/C+) (Fig. 2a). In the involution group tumors, overall immune cell (CD45+) content (Fig. 2b) increased compared to nulliparous tumors. Within this CD45+ compartment, we observed relative increases in immature monocytes (Fig. 2c) with an almost equal reciprocal decrease in mature macrophages (Fig. 2d). This increased presence of immature monocytes in tumors experiencing the involution compared to nulliparous microenvironment is consistent with a groundswell of literature in recent years correlating the presence of immature myeloid cells (MDSCs and monocytes) with poorer tumor outcomes and impaired anti-tumor T cell immunity [38, 44–46].

### Involution reduces intratumoral T cell accumulation

**Fig. 3:**
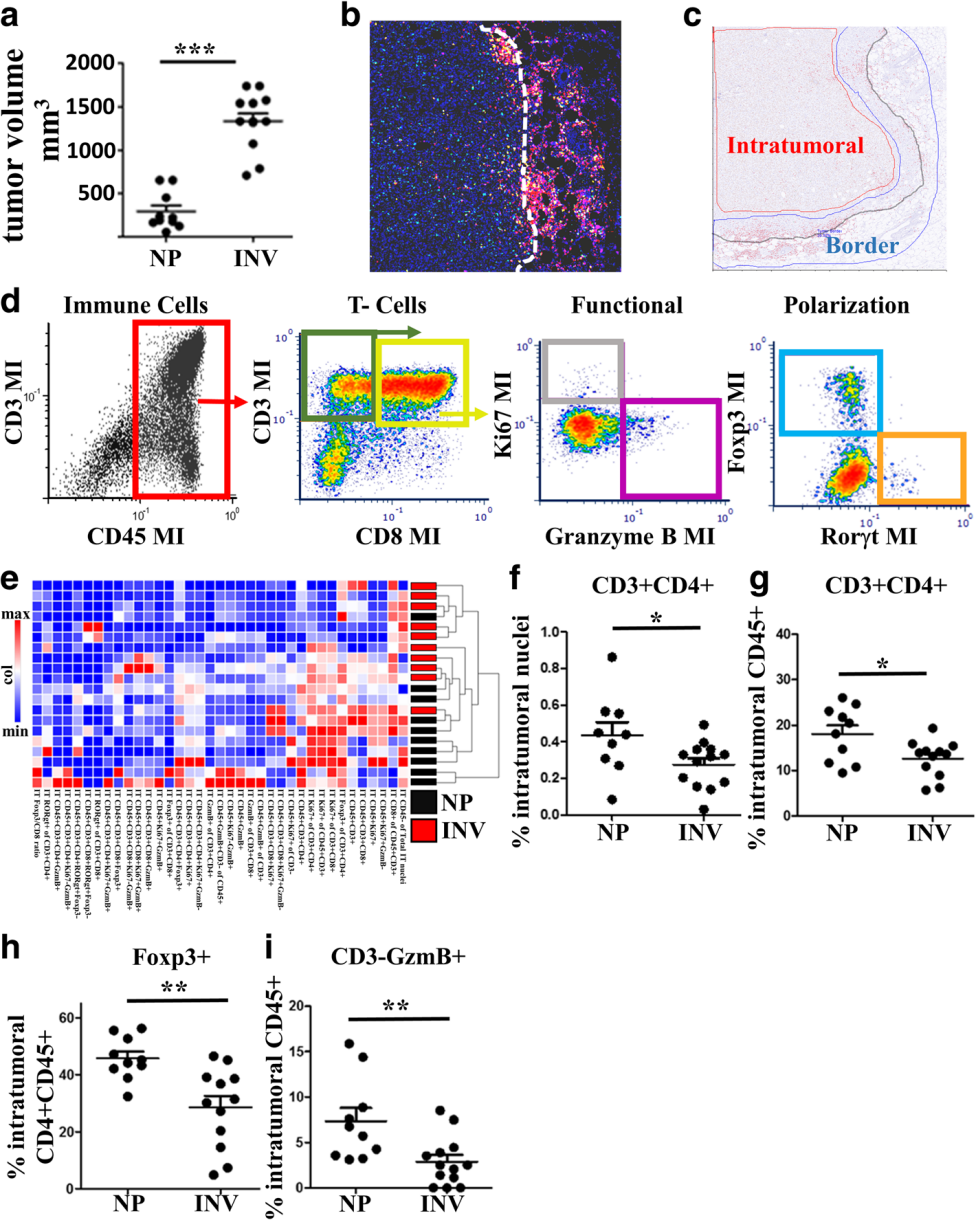
Seven antibody multiplex IHC analysis to characterize T cell composition of nulliparous and involution group tumors. A subset of study tumors **a** of representative size, growth and variation observed in the entire involution tumor cohort were evaluated for CD45, CD3, CD8, Ki67, Foxp3, Rorγt, and GzmB using multiplex IHC. **b** Representative image of INV group tumor where immune cell IHC signals have been pseudo-colored (all colors other than blue nuclei) illustrates an enrichment of immune cells at the tumor border (dashed white line). **c** Multiplex IHC signal was segmented based upon signal location relative to the tumor border (black line), with tumor border region (area between blue lines) and intratumoral regions (area inside red line) analyzed separately. **d** Representative image cytometry gating schema for T cell populations of interest evaluated in bulk by **e** hierarchical clustering of intratumoral lymphocytes and individually, showing a decrease in involution tumors of intratumoral CD3 + CD4+ T cells as proportion of **f** all intratumoral cells and **g** intratumoral CD45+ cells, with **h** Foxp3 + CD4+ T cells reduced as compared to all intratumoral CD3 + CD8-(putative CD4+) T cells, and **i** CD45 + CD3-Gzmb+ immune cells are decreased relative to intratumoral CD45+ cells during involution as well. Determination of statistical differences between nulliparous (NP) and involution (INV) were based upon two-tailed Student’s t-test comparison with *p* < 0.05 (*), p < 0.01 (**), and *p* < .001 (***)

To address T cell impairment in the involution group tumors, we first employed a recently developed technological advancement, quantitative multiplex IHC [41], on a representative subset of tumors (Fig. 3a). The advantage of this approach is it allows for registry of multiple antibody based signals to a single cell, permitting subsequent immune cell population identification and quantitation analogous to flow cytometry while maintaining context of the tissue’s architecture (Fig. 3b, c, d). Tissue contextual characterization is particularly important for adaptive immune cells, as factors that contribute to T cell impairment include not only activation and abundance but also restriction in trafficking. In particular, factors that control T cell accumulation at the tumor border are different from those that permit tumor penetration. Each adaptive immune locale preferentially provides either impedance of tumor cell outgrowth and dissemination (tumor border) or tumor clearance (intratumoral). In our model, tumor border accumulation of immune cells is evident (Fig. 3b & c, tumor border region highlighted in blue) as well as sparse presence of intratumoral immune cells. These data are consistent with numerous reports in the literature demonstrating differential regulation of tumor border from intra-tumoral T cell accumulation [47–50] and highlight the importance of spatially discerning quantification.

Using multiplex IHC we first delineated between the intratumoral and tumor border regions and then assessed the composition and character of T cells in these respective tumor compartments (Fig. 3d). Hierarchical clustering of parameters derived from this 7 antibody multiplex IHC analysis revealed less defined clustering and statsitically insignificant differences between nulliparous and involution tumors at the tumor border (Additional file 1: Figure S1a, Figure S1b). However, cluster analysis of the intra-tumoral immune cells (Fig. 3e) revealed tumor differences in immune components between the involution and nulliparous groups. Significant intratumoral differences observed were reduced overall numbers of intratumoral CD4+ T cells (Fig. 3f), and numbers of CD4+ T cells as a fraction of CD45+ (Fig. 3g) cells in the involution group tumors. When investigating the character of the intra-tumoral CD4+ T cells by examination of transcription factors involved in T cell helper/effector polarization, we unexpectedly observed a decrease in the relative abundance of Foxp3+ T cells (Fig. 3h) in the involution group tumors. Although different, levels of Foxp3+ CD4+ T cells in both the nulliparous (NP) and involution (INV) tumors remained very high (45% and 30%, respectively, of the CD4+ T cell population) compared to previously reported levels of regulatory T cells in the lymph node and spleen of non-tumor bearing animals. These elevated levels of Foxp3+ T cells are consistent with tumor associated impairment of anti-tumor immunity in both groups. Lastly, we observed a significant reduction in CD45 + CD3- Granzyme B+ cells in the intratumoral regions of involution-group tumors (Fig. 3i). While not definitive, this phenotype is consistent with reduction in NK cells, which are reported to provide some level of cellular immunity against tumors [51, 52]. Collectively, these observations broadly illustrate that the transient ~7-day event of mammary gland involution has a durable tumor promotional imprint on both innate and adaptive immune cells in the resulting tumor microenvironment.

### Ibuprofen reduces tumor burden in immune competent hosts

Glandular expression of the enzyme COX-2 is dynamically upregulated during the involution window. If COX-2 is active, the resulting prostaglandins would be predicted to have potent tumor promotional effects on both innate and adaptive immune cells through the classical mechanisms described earlier. For these reasons we sought to determine whether PGE2 or other COX-2 mediated arachidonic acid metabolites were expressed at elevated levels in the mammary gland during involution and could be effectively targeted with ibuprofen administration.

**Fig. 4:**
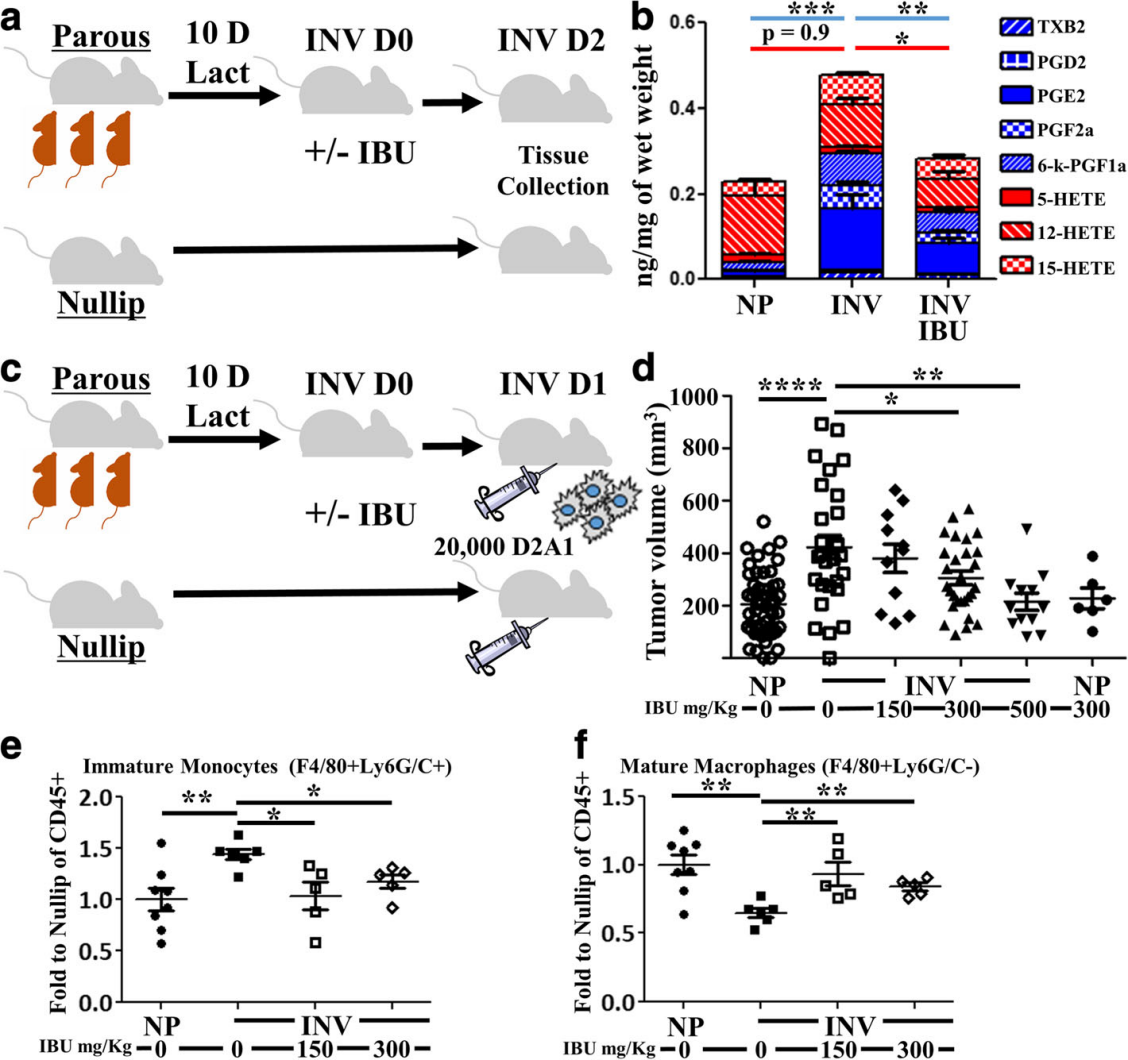
Ibuprofen impairs mammary prostaglandin production, tumor promotion and immature myeloid cell frequency during involution. **a** INV group mice were generated by breeding, and pups nursed for 10 days (10 D). Pups were removed to initiate synchronous mammary gland involution at which time ibuprofen containing (IBU) chow (300 mg/kg chow) or normal chow was administered to INV mice. Control NP mice received normal chow only. Two days later (INV D2 for INV group mice) mammary glands were harvested and **b** assessed by lipid mass spectroscopy for absolute quantification of the indicated prostaglandin (blue) and leukotriene (red) family members. **c** The above breeding schema was repeated. Mice received the indicated dose of ibuprofen starting at initiation of involution and in age-matched NP mice. One day later, mice received an orthotopic fat pad implantation of 20,000 syngeneic D2A1 mammary epithelial tumor cells. At ~ 3 weeks post injection **d** tumor sizes were recorded. The tumor data are an aggregate of three independent experiments. Data were normalized between experiments by utilizing the time point where the mean tumor volume of the NP group tumors was the same for all three studies. Tumors were digested into single cell suspensions for determination of **e** immature monocyte and **f** mature macrophage content by flow cytometry. Determination of statistically significant differences were based upon two-tailed Student’s t-test between the indicated groups with p < 0.05 (*), p < 0.01 (**), p < .001 (***), *p* < .0001 (****)

To this end, prostaglandin and leukotriene levels were evaluated by lipid mass spectroscopy from the mammary glands of nulliparous (NP) controls and mice at 48 h of involution (InvD2) without and with 300 mg ibuprofen/kg chow, the dose equivalent of a 150 pound person consuming 250 mg ibuprofen daily (Fig. 4a). In InvD2 mammary glands, we observed a significant increase in the production of many of the COX-2 generated prostaglandins (Fig. 4b, blue bars) compared to those observed in age matched, nulliparous controls. Notably, PGE2 was the most upregulated prostaglandin in the involuting mammary gland. At the same time we observed that global leukotriene levels (red bars) remain unchanged with involution; however, individual leukotrienes, which are products of non-COX mediated arachidonic acid metabolism, modestly changed between nulliparous and involution. In involution mice that received ibuprofen, significant reductions in both prostaglandins and leukotrienes were observed compared to involution mice eating standard chow.

Knowing that prostaglandins, including PGE2, are elevated during involution and targetable with ibuprofen, we sought next to test if ibuprofen blockade of COX-2 would impair involution associated tumor growth. Involution group mice received chow with either 0, 150, 300 or 500 mg ibuprofen/kg of chow. A cohort of nulliparous mice were also given chow with 300 mg ibuprofen/kg. Twenty-four hours later mice were orthotopically injected with 20,000 D2A1 syngeneic Balb/c mammary tumor cells into the mammary fat pad and monitored for tumor growth (Fig. 4c).

An aggregate of 3 separate studies showed that tumor cells injected into the involution mammary microenvironment had significantly increased overall tumor size in comparison to nulliparous controls (Fig. 4d). Furthermore, ibuprofen reduced tumor volume in the involution group in a dose dependent manner, with significant reductions in tumor volume in animals receiving 300 mg and 500 mg ibuprofen/kg chow. Importantly, in nulliparous animals, the administration of 300 mg ibuprofen/kg chow did not impair tumor growth compared to nulliparous animals on normal chow. This observation is not consistent with ibuprofen meditating its effects entirely through tumor-cell intrinsic properties. Instead our data are most consistent with ibuprofen targeting the prostaglandin rich microenvironment of the involuting mammary gland, an environment not present in the homeostatic nulliparous gland.

**Fig. 5:**
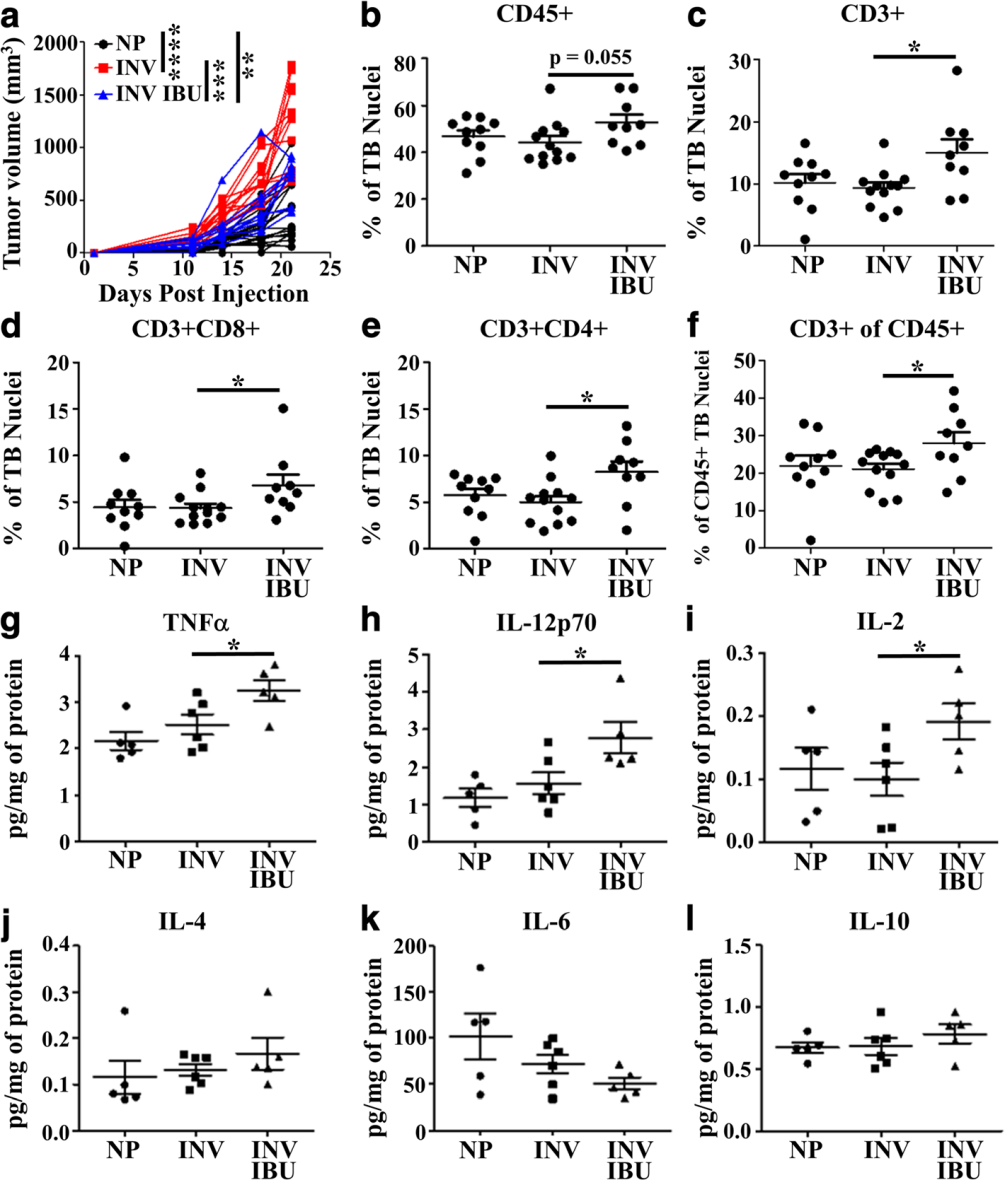
Ibuprofen enhances tumor border T cell abundance and intratumoral Th1/M1 related cytokines. A subset of study tumors **a** representative of tumor growth from their respective groups; involution (INV), involution with 300 mg/kg ibuprofen (INV IBU) and nulliparous (NP), were stained for immune cell populations using multiplex IHC and data analyzed by image cytometry. Abundance of **b** CD45+, **c** CD3+, **d** CD3 + CD8+, and **e** CD3 + CD4 + (CD8-) as percent of total nuclei at the tumor border (TB), and composition of **f** CD3 + CD4 + (CD8-) relative to the CD45+, demonstrate ibuprofen effects on T cell populations at the tumor border. **g-l** Frozen tumor samples were subjected to cytokine level detection to determine intratumoral protein levels of **g** TNFa, **h** IL-12p70, **i** IL-2, **j** IL-4, **k** IL-6, and **l** IL-10. Determination of statistically significant differences were based upon two-tailed Student’s t-test between the indicated groups with *p* < 0.05 (*)

One potential mechanism by which ibuprofen treatment during involution might suppresses tumor growth is through the restoration of an anti-tumor immune response. Supportive evidence for this mechanism is suggested by flow cytometry analysis of tumors from involution mice consuming 150 or 300 mg ibuprofen/kg chow. Ibuprofen treatment resulted in a decrease in the number of immature monocytes and a concomitant increase in mature macrophages (Fig. 4e & f). To assess tumor associated T cells, we utilized a representative subset of mammary tumors from nulliparous, involution and involution animals receiving 300 mg ibuprofen/kg chow (Fig. 5a compared Fig. 4d). In this multiplex IHC analysis, we found that ibuprofen induced the most dramatic immune alterations at the tumor border with a trend for increased CD45+ cells (Fig. 5b), and significant increases in total CD3+, and CD8+ and CD4+ T cell compartments (Fig. 5c-e). This increase in tumor border T cells was not merely a consequence of an overall increase in immune cells, as T cell composition expressed as a relative percent of CD45 was also significantly increased (Fig. 5f).

We next sought to determine if ibuprofen treatment, in addition to impacting immune cell abundance, impacts cytokine production as an indirect measure of immune cell polarization. Whole tumor cell lysates were probed using a multiplex antibody-based affinity protein cytokine array. In ibuprofen treated involution group tumors we observed an immune milieu enriched for TNFα (Fig. 5g) and IL-12p70 (Fig. 5h), cytokines associated with mature M1 macrophage polarization and known to facilitate Th1 and cytotoxic T cell function. We also observed an increase in IL-2, a potent mediator of T cell proliferation and survival (Fig. 5i). This cytokine milieu is consistent with the multiplex IHC observation of increased T cells along the tumor border with ibuprofen treatment (Fig. 5c-f). Importantly, increases in TNFα, IL-12p70 and IL-2 are not merely consequent of enhanced immune cell presence, as a number of other cytokines were unaltered by ibuprofen treatment including those associated with tumor associated macrophages, monocytes and M2 polarized macrophages [IL-4 (Fig. 5j), IL-6 (Fig. 5k), IL-10 (Fig. 5l)]. Having observed these changes in the polarized immune milieu and T cell numbers in the tumor we next asked whether involution and ibuprofen influences immunity beyond the tumor itself.

**Fig. 6:**
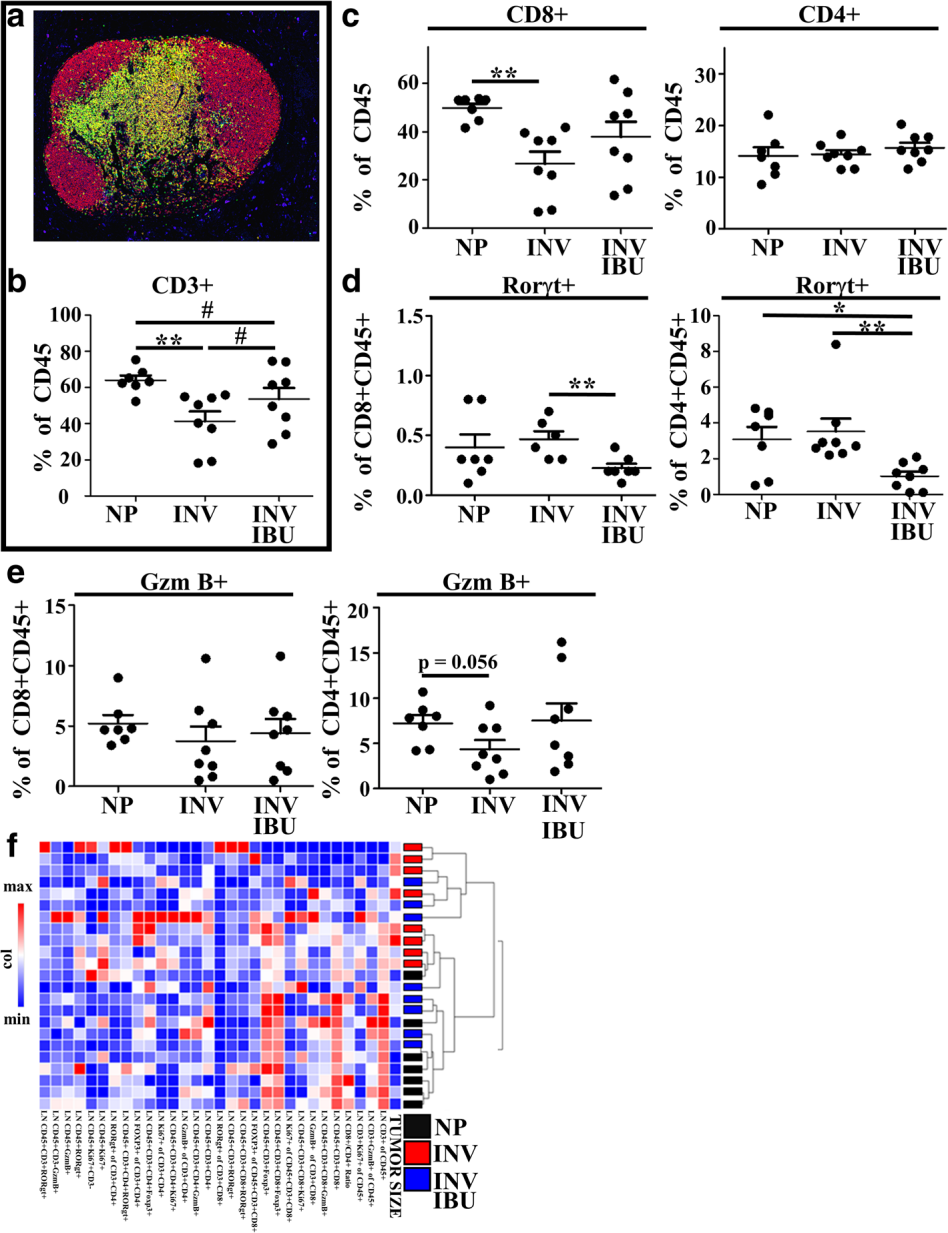
Ibuprofen enhances tumor draining lymph node T cell abundance and cytotoxic programming in mice. **a** Representative pseudo-colored composite of a T cell centric 7 antibody multiplex IHC image of tumor draining lymph node from an INV group mouse at 3wk post tumor injection (study end). CD45 is shown in red, CD3 in yellow, CD8 in green, Foxp3 in orange, GzmB in purple, Rorgt in light blue, and nuclei in blue. Quantitation of multiplex IHC signals of tumor draining lymph node to determine **b** CD3+ proportion of CD45+ cells, **c** CD3 + CD8+ and CD3 + CD4+ proportion of CD45+ cells, **d** Rorγt + proportion of CD8 + CD3 + CD45+ and CD4 + CD3 + CD45+ cells, and **e** Granzyme B (GzmB) + proportion of CD8 + CD3 + CD45+ and CD4 + CD3 + CD45+ cells. **f** Hierarchical clustering of lymph node multiplex IHC data show an association of evaluated parameters with tumor size and tumor cohort. Determination of statistically significant differences were based upon two-tailed Student’s t-test between the indicated groups with p < 0.05 (*) and p < 0.01 (**)

Since tumor T cell helper and effector differentiation is predominantly thought to occur in the tumor draining lymph node we evaluated whether, within the node, involution itself or in combination with ibuprofen, impacts T cell number and phenotype. Using multiplex IHC (Fig. 6a), we examined relative T cell abundance, differentiation, and effector function. Strikingly, in the tumor bearing involution group, we observed involution to dramatically reduce the relative T cell abundance of the lymph node (Fig. 6b). In contrast, administration of ibuprofen restored CD3+ T cell numbers (Fig. 6b). The involution specific decrease in lymph node T cells was specifically attributable to a loss of CD8+ T cells, not CD4+ T cells (Fig. 6c). We also observed that administration of ibuprofen significantly reduced the frequency of Rorgt+ T cells in both CD4+ and CD8 + T cell compartments (Fig. 6d). This is noteworthy as Rorgt+ T cells are programmed by milieu influences of TGFbeta and IL-6, key cytokines known to be enhanced during involution and generally considered suppressive of anti-tumor immunity.

Finally, Granzyme B has garnered attention as a key effector molecule in T cell cytotoxicity, and both CD4+ and CD8+ T cells can express granzyme B to kill cells expressing foreign and aberrant self-antigens [53, 54]. Within the tumor draining lymph node, we observed significant decreases in granzyme B expression within the CD4+ compartment in the involution group, with granzyme B restored upon ibuprofen treatment. We observed similar, although not significant, trends in the CD8+ T cell compartment (Fig. 6e). Based upon previous work by our lab and others, the global impact of ibuprofen treatment during involution likely involves additional mechanisms outside of the adaptive immune response. Nonetheless, hierarchal clustering analysis of the T cell centric multiplex IHC panel for the lymph node (Fig. 6f) clustered well by overall tumor size (most right column) and study group (black versus red), suggesting a role for the lymph node biology in determining tumor burden. Collectively, the data thus far illustrates that involution has durable influences on the tumor immune milieu, altering leukocyte composition and polarization in the directions of a pro-tumor microenvironment. In contrast, ibuprofen administration during involution promotes anti-tumor immunity.

### Ibuprofen impacts normal involution associated macrophages and monocytes

Our above in vivo studies identify an impact of ibuprofen on the tumor immune microenvironment in the involuting mammary gland. We next wanted to assess if ibuprofen impacts the immune microenvironment of involuting mammary gland in the absence of tumor. We focused on involution associated immature monocytes as they are dramatically elevated in the early time points of involution, when tumor introduction occurs in our model. Further, in the tumor microenvironment, it is the analogous immature monocytes that give rise to tumor associated macrophages with “M2”-like character, and which have previously been observed to be elevated during involution and contribute to the pro-tumorigenic immune milieu of the normal involuting mammary gland [18]. These considerations provided rationale for examining gene expression changes in response to ibuprofen for both immature monocytes and macrophages from the non-tumor bearing gland.

**Fig. 7:**
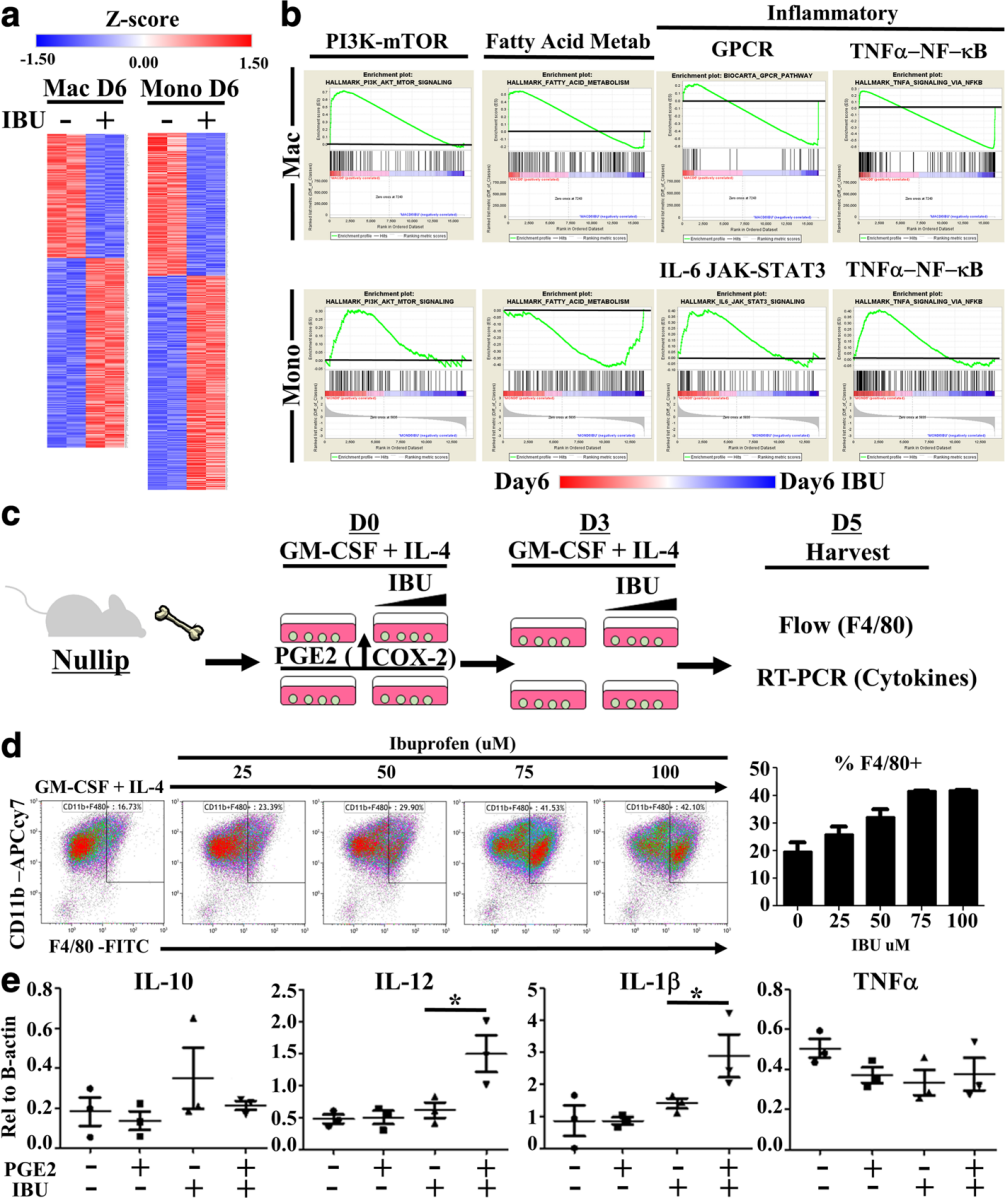
Involution associated macrophages and monocytes are targets of ibuprofen. Immature monocytes and macrophages from involution day 6 (D6) mice fed 300 mg/kg ibuprofen chow (+) or normal chow (−) were flow sorted based upon expression level of F4/80 and lineage negative marker. RNA was purified and subjected to whole exome RNA sequencing. **a** Significantly differentially expressed genes were determined based upon fold change and *p*-value distribution for Macrophages (Mac) and Monocytes (Mono) separately and graphically represented as relative z-score gene expression by gene (row). **b** Whole gene sets for macrophages (top row) or monocytes (bottom row), each with and without ibuprofen, were subjected to Gene Set Enrichment Analysis using publically available gene sets. The highly enriched gene sets for PI3K-mTOR, fatty acid metabolism, GPCR, TNFα-NFkB, and IL-6 Jak-STAT3 G protein-coupled receptor (GPCR), were selected for graphical representation. Black lines denote level of zero enrichment. Peaks or valleys that occur on the edges represent pathways differentially regulated by ibuprofen. **c** Bone marrow monocyte cultures were established by incubation of bone marrow cells from nulliparous animals in the presence of GM-CSF (20 ng/mL) and IL-4 (10 ng/mL) with increasing concentrations of ibuprofen for five days (D5) with or without an initial introduction of PGE2 (.92 ng/mL). Media was replenished with fresh ibuprofen where indicated on day 3 (D3). Day 5 adherent and non-adherent cells were collected for evaluation of monocyte maturation by **d** flow cytometry evaluation of CD11b and F4/80 expression and **e** RNA expression of cytokines relative to b-actin by RT-PCR. Comparisons of statistically significant differences were based upon two-tailed Student’s t-test between the indicated groups with *p* < 0.05 (*)

To this end, at 6 days post involution, mammary gland digests were flow sorted based upon expression of lineage markers and F4/80 positivity, analogous to the flow characterization employed in Fig. 2. Further flow characterization demonstrated expression of F4/80 was limited to two CD11b + immune cell populations with distinct F4/80 and Ly6C expression levels concordant with differential activation profiles (Additional file 2: Figure S2a). We therefore defined F4/80 low but not negative cells as immature monocytes and F4/80 high cells as mature macrophages. From these cells, RNA was isolated and selected based upon 3’ Poly A bead capture to examine only exon encoded protein expressing genes by RNA sequencing. Principal component analysis based upon the RNA expression profiles distinctly separated F4/80 high (putative macrophages) from F4/80 low (putative monocytes) cell types (Additional file 2: Figure S2b). In both populations, ibuprofen treatment resulted in differential gene expression patterns (Fig. 7a and Additional file 2: Figure S2c).

Gene Set Enrichment Analysis of the full gene sets showed ibuprofen altered pathways involved in basal energy metabolism, fatty acid metabolism and specific axes of macrophage and monocyte activation (Fig. 7b). Ibuprofen induced gene expression changes in immune activation pathways involving GPCR and IL-6, results consistent with well documented biology of PGE2 signaling through G-protein coupled receptors and downstream IL-6 production. In addition, ibuprofen treatment differentially impacted monocyte and macrophage TNFa/NF-kB pathways. These data aligned with the established paradigm of PGE2 impairing TNFa production by myeloid cells and are consistent with our observations of elevated TNFa in involution associated tumors upon treatment with ibuprofen (Fig. 5g). Overall, these pathway differences synchronize conceptually with the biology of the involution microenvironment that is rich in lipid, promotes myeloid cell recruitment, and favors myeloid cell activation. Most importantly, these gene expression observations further support the idea that monocytes and macrophages are targets of ibuprofen in the involution microenvironment regardless of tumor presence.

To address whether ibuprofen directly targets monocytes and macrophages we employed a bone marrow derived monocyte cell culture system. Since our in vivo data revealed ibuprofen to be associated with monocyte differentiation, we utilized GM-CSF + IL-4, cell culture conditions that favor production of immature myeloid cells over mature macrophages [55, 56]. To more closely resemble the involution environment, PGE2 was added into one set of wells. We found that PGE2 induces the expression of COX-2 (Additional file 3: Figure S3a), kick-starting a feedforward cycle of prostaglandin production and thus modeling aspects of involution in which COX-2 is elevated. After a total of 5 days of culture, both adherent and non-adherent cells were then harvested, pooled, and evaluated for macrophage differentiation markers CD11b and F4/80 by flow cytometry and differential cytokine gene expression by RT-PCR (Fig. 7c).

We observed that addition of ibuprofen enriched macrophage differentiation in a dose dependent manner (Fig. 7d). We next confirmed that differential cell survival did not account for the increased fraction of mature macrophages with ibuprofen treatment, as assessed by MTT assay. Only at higher doses of ibuprofen were modest decreases in cell viability observed (Additional file 3: Figure S3b). We next evaluated for M1 and M2 associated cytokine mRNAs from the 5-day cultures and observed that the initial administration of PGE2 followed by COX1/2 blockade with 100 μM ibuprofen treatment greatly enhanced IL-12 and IL-1beta gene expression (Fig. 7e). In total, these observations demonstrate that ibuprofen treatment enhances M1 biased macrophage differentiation in a PGE2 rich microenvironment. This is consistent with results from others showing that ibuprofen diminishes MDSC overall abundance and M2 associated cytokines (IL-4/IL-10) while facilitating IL-12 production [57–60].

Collectively our data depict tumors arising in the involution environment as enriched in immature myeloid cells and having impaired T cell responses. We found that administration of ibuprofen during involution reverses these trends, promoting more fully differentiated macrophages and an immune milieu consistent with M1/Th1 immunity. To consider whether the ibuprofen induced changes in immune cell phenotype and gene expression have any correlation to breast cancer outcomes in humans, we interrogated the TCGA breast cancer data set. We generated Kaplan-Meier survival plots based upon gene expression of key molecules found to be differentially regulated by ibuprofen in our mouse studies.

**Fig. 8:**
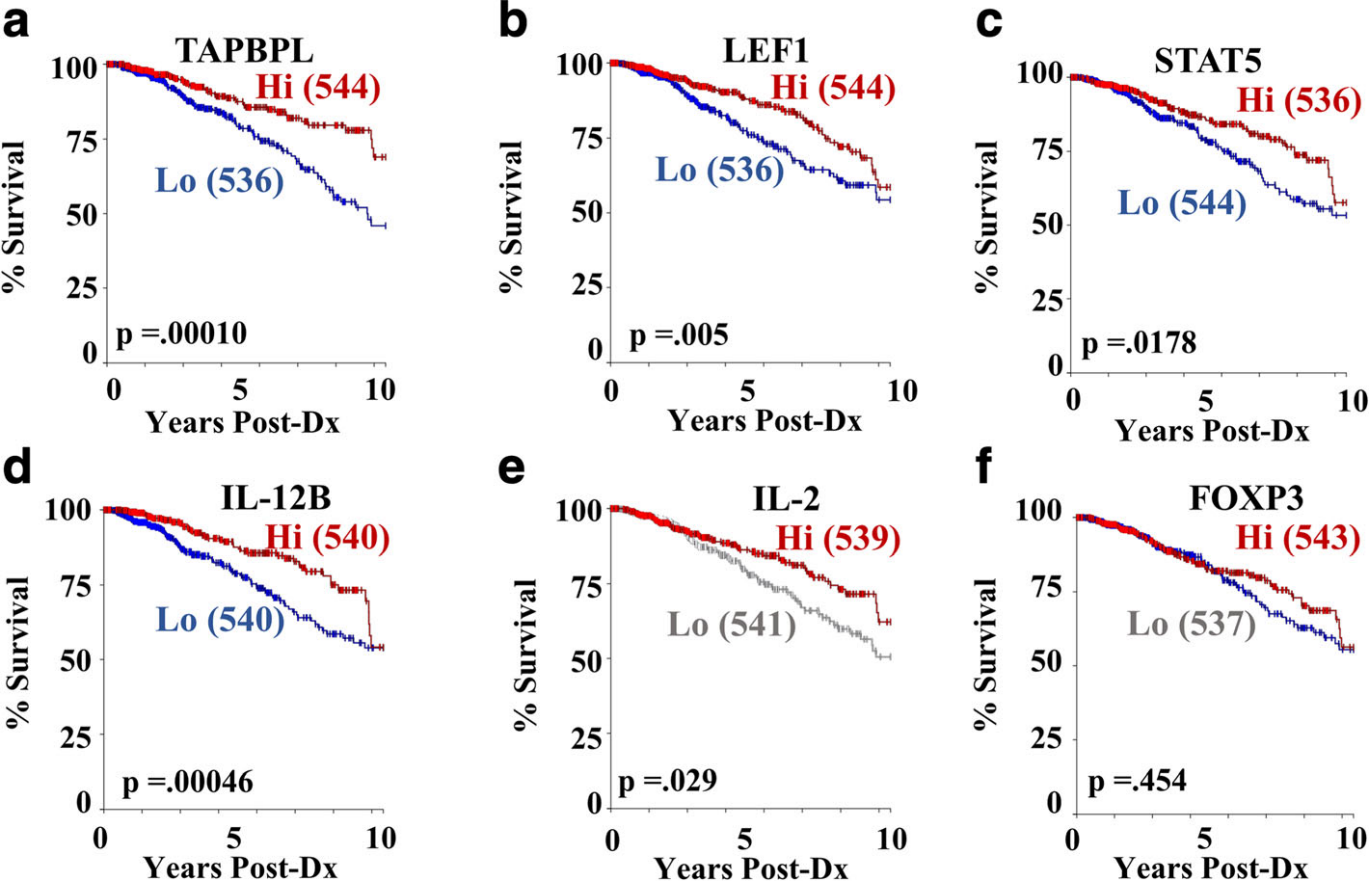
Ibuprofen related genes predict survival in humans TCGA Breast Cancer Data Set. Genes identified from differential expression analysis of involution macrophages, monocytes, and tumors with and without ibuprofen treatment in the above mouse studies were input as genes of interest for determination of correlation with survival in human breast cancer. The ibuprofen regulated genes **a** TAPBPL, **b** LEF1, **c** STAT5, **d** IL-12 and **e** IL-2 and **f** FOXP3 associate with better prognosis as visualized in the Kaplan-Meier plots derived from the TCGA-Breast Cancer data sets. The numbers of cases with high (Hi-red) and low (Lo-blue or gray) gene expression is indicated in parentheses

The first gene we assessed was Tapbpl, a gene upregulated with ibuprofen treatment in macrophages at day six of involution (Fig. 7a and Additional file 2: Figure S2c) and involved in endoplasmic reticulum facilitation of MHC/antigen presentation. Interestingly, we found that Tapbpl high expression significantly correlates with better survival outcomes (Fig. 8a). Likewise, LEF1 and STAT5 related genes were highly expressed in ibuprofen treated myeloid cells (Additional file 2: Figure S2d) and highly correlated with better outcomes in the TCGA primary breast cancer data set (Fig. 8b & c). Lastly, in this report, we have repeatedly remarked on the influence of ibuprofen treatment on increasing IL-12 and IL-2 levels or T cell numbers (Figs. 3g, 5h, i, and 7e). In examining the TCGA data set, we see again that these ibuprofen upregulated molecules (IL-12 Fig. 8d and IL-2 Fig. 8e) have strong prognostic value for overall survival in the TCGA breast cancer human cohort.

We noted previously that contrary to our initial hypothesis, involution associated tumors displayed a significant overall decrease in intratumoral Foxp3 + CD4+ T cells (Fig. 3g). In most immunocompetent tumor models and data from multiple human studies, such a decrease would be associated with enhanced anti-tumor immunity and reduced tumor growth. To examine if this finding in our mouse model represented an important disjunction from human breast cancer, we once again interrogated breast cancer outcomes based upon FOXP3 gene expression in the TCGA data set. Surprisingly, yet consistent with our rodent models, we found no difference in 10 year survival between FOXP3 high and FOXP3 low patients (Fig. 8f), indicating the presence of regulatory T cells, as defined based upon the sole expression of FOXP3, may have no observable prognostic significance in breast cancer outcomes.

In sum, these data identify a role for ibuprofen in mitigating the tumor promotional microenvironment of the involuting mammary gland through the promotion of anti-tumor immunity. Thus, upon first consideration, and given that mammary gland involution is only a transient tumor promotional window, it might seem rational to move forward with the recommendation to administer ibuprofen to women at time of weaning. However, while PPBC is associated with very poor outcomes regardless of tumor subtype it is also relatively rare, occurring in less than 0.5% of postpartum women. Further, given that we have identified ibuprofen administration during involution as promoting Th1/Tc1 differentiation, we thought it important to address whether ibuprofen treatment could result in negative sequelae, such as instigation of autoimmunity or decreases in reproductive health. In fact, it has been demonstrated that inflammatory adjuvanted (Complete Freund’s adjuvant) immunization of mice to the transient antigens of lactation can result in lactation failure and consequently failure of pups to thrive [61]. Furthermore, impairment of endogenous tolerance mechanisms evoked during involution have been documented to result in persistent morphological aberrations of the gland including increased fibrosis and epithelial cell hyperplasia [21, 62]. Thus, determining the safety of ibuprofen is an essential initial first step towards consideration of ibuprofen as a safe chemopreventive agent for young women’s breast cancer.

**Fig. 9:**
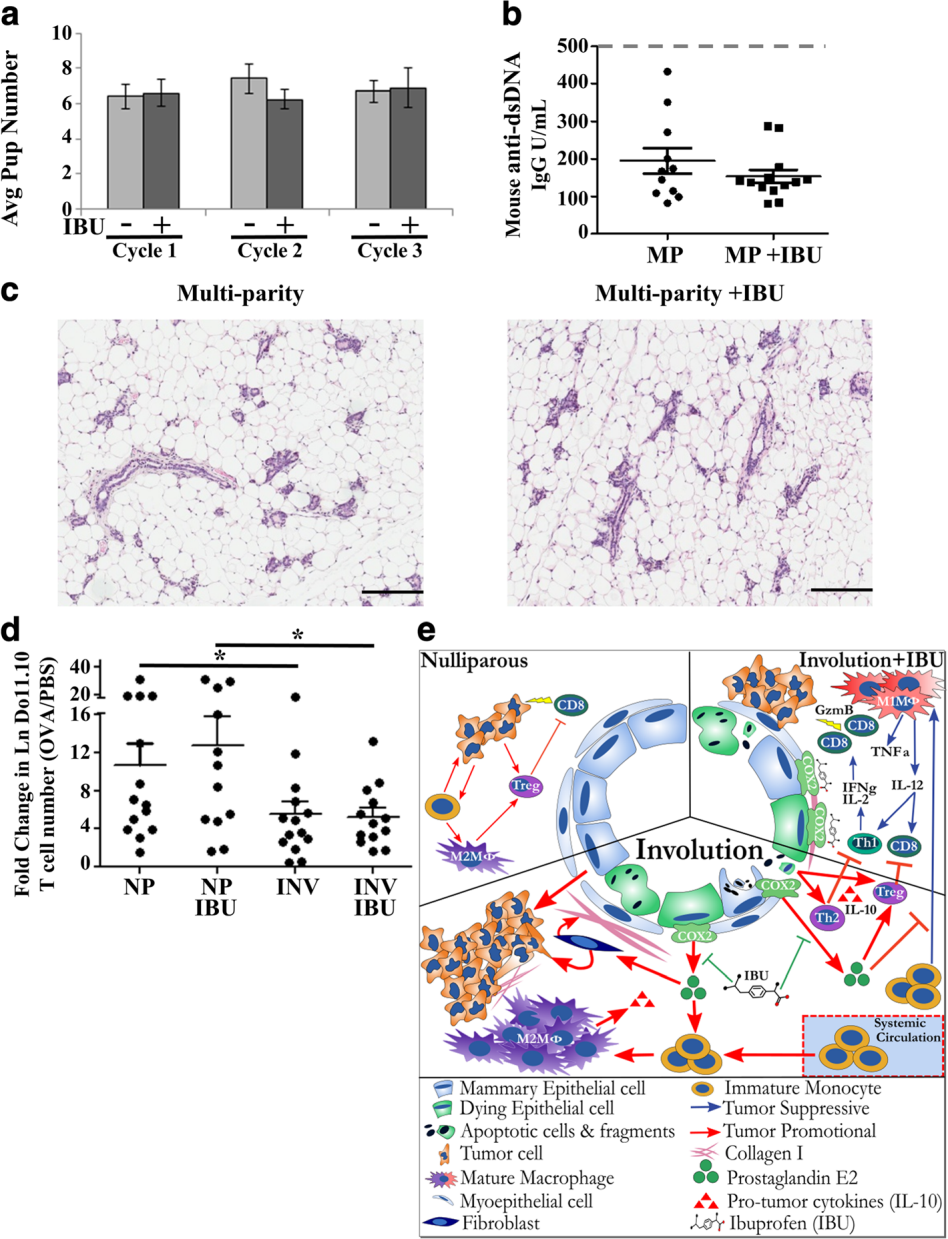
Ibuprofen treatment of non-tumor baring involution hosts has no observed impact on reproductive success or immune reactivity. **a** Numbers of pups born and successfully nursed for 10 days (start of synchronized mammary gland involution) at each cycle of breeding with (+) or without (−) 10 days of ibuprofen (IBU) after each round of weaning-induced involution (*n* = 10–15 mice/group). **b** Assessment of anti-dsDNA antibodies in the circulating maternal blood after completion of the third pregnancy/lactation/involution cycle. Maximum linear range of anti-ds-DNA detection is 1000 IgG U/mL, and gray dashed line indicates level of anti-dsDNA antibodies commonly observed in spontaneous autoimmune mouse serum. **c** Representative H&E sections from mice after completion of the third pregnancy/lactation/involution cycle (Multi-parity) in the presence or absence of 10 days of ibuprofen (IBU) after each round of weaning-induced involution. **d** Assessment of antigen specific naïve T cell activation. Assay was established as outlined in Additional file 4: Figure S4. Antigen specific T cell activation is expressed by fold change in absolute number of antigen specific Do11.10 T cells in the draining lymph node of ova injected to PBS control in nulliparous (NP) or involution (INV) mice with or without ibuprofen (IBU). Determination of statistical differences between groups was based upon one-tailed Student’s t-test according to previously published results with this assay, p < 0.05 (*). **e** graphical summary reported results

To begin to address these concerns, Balb/c mice were bred and nursed for 10 days as described previously. Upon pup removal, one group of mice received ibuprofen chow (300 mg/kg chow) for 14 days. This schema of breeding and treatment was repeated for 2 more subsequent cycles with the same mice to determine if serial ibuprofen administration during involution negatively impacts normal mammary gland involution, subsequent breeding and lactation success, or promotes autoimmunity. Firstly, we found that ibuprofen group mice had the same reproductive success as control mice, as evidenced by similar pregnancy schedules, pup number per pregnancy, and pup survival (Fig. 9a and data not shown). Five weeks after the third weaning cycle, mice were euthanized, blood collected to evaluate for the presence of anti-dsDNA antibodies, an indicator of autoimmunity induction, and mammary glands collected to assess changes in gland histology. No evidence of elevated anti ds-DNA antibodies from circulating maternal blood (Fig. 9b), nor alterations in glandular morphology (Fig. 9c), were observed differentially between ibuprofen treated and untreated multiparous mice.

These observations support a lack of induced autoimmunity in non-tumor bearing hosts in response to repeated ibuprofen administration during the involution window. However, we were concerned that the level of immune activation required to generate detectable double-stranded DNA antibodies, or grossly impair gland morphology and pup rearing, may be insufficient measures of potential host detriment. Specifically, ibuprofen could still be eliciting autoreactive M1/Th1/Tc1 like inflammatory responses during involution that might negatively impact the host but occur below the sensitivity afforded by these endpoints. Recently, utilizing a highly sensitive antigen-specific adoptive T cell transfer assay, we reported that normal mammary gland involution employs a regional antigen-specific tolerogenic mechanism for T cell suppression [19]. We employed this assay to sensitively evaluate both number and programming of T cells in response to antigen exposure in the mammary gland during involution, in the absence and presence of ibuprofen treatment.

In this assay (Additional file 4: Figure S4), purified Balb/c TCR transgenic derived, naïve ova antigen-specific splenic T cells (Do11.10) are transferred into nulliparous and involution female Balb/c hosts in which mice are given control or ibuprofen (300 mg/kg) containing chow. Two days later, whole purified chicken ovalbumin is introduced into one mammary gland while PBS only is administered in the contralateral gland. Five days later, mammary gland draining inguinal lymph nodes are harvested and T cell number and transcription factor programming profile determined by flow cytometry. To account for intra-animal variations in T cell engraftment, we utilize the ratio of absolute numbers of transgenic T cells from the antigen exposed side compared to the PBS side as indicator of naive T cell activation (Fig. 9d). Consistent with what we have previously published, we observed a significant decrease in the ability of T cells to increase in cell number in response to antigen in the involuting host. In mice who received ibuprofen, we likewise observed the same decrease in antigen specific T cell number as observed in involution mice who did not receive ibuprofen (Fig. 9d). Lastly, ibuprofen had no benefit or detriment to T cell numbers in nulliparous hosts (Fig. 9d). These data further support the notion that ibuprofen treatment during involution does not increase risk of auto-antigen reactivity during mammary gland involution in the absence of tumor.

## Discussion

Elevation of COX-2 activity is reported in greater than 40% of all breast cancers [63] and preclinical data supporting the efficacy of NSAIDs in suppressing breast cancer has made COX-2 a logical target for breast cancer therapy and chemoprevention. Here we investigated the role of COX-2 as a potential target in postpartum breast cancer, a poor prognostic form of breast cancer driven in part by a tumor promotional microenvironment present within the involuting mammary gland. Utilizing a fully immune competent rodent model of weaning-induced mammary gland involution, we demonstrate that the microenvironment of the involuting mammary gland is actively enhanced for prostaglandin production downstream of COX-2. Furthermore, through targeting of COX-2 by low dose ibuprofen administration, we reveal COX-2 to be a significant tumor promotor in the involuting mammary gland. Importantly, we demonstrate that involution and ibuprofen elicit distinct and opposing myeloid and T cell attributes in the resulting tumor microenvironment. Specifically, we find that involution promotes increased intratumoral numbers of immature myeloid cells, decreased numbers of mature intratumoral macrophages, and decreased tumor associated and tumor draining lymph node residing T cells, compared to nulliparous animals. In contrast, ibuprofen treatment during involution increases the abundance of mature macrophages, T cells, and tumoral cytokines (IL-2, IL-12, TNFa) supportive of Th1 and anti-tumor immunity (Fig. 9e).

Furthermore, we show that ibuprofen administration during involution without the presence of tumor does not result in overt instigation of autoimmunity, impact the physiologically normal T cell suppression that occurs during involution, effect weaning-induced lobular regression, or reduce the ability of treated dams to successfully raise pups from a subsequent pregnancy. Collectively these observations suggest ibuprofen administration could act as a safe and effective chemo preventive immunotherapy during involution. Further, our data reveal a potential benefit of NSAIDs in the treatment of postpartum breast cancer patients.

Recent spectacular yet sporadic results employing immunotherapy strategies in human and mouse studies of solid tumors have highlighted the immune system as a critical determinant in the tumor microenvironment [64, 65]. Many studies have specifically drawn attention to the presence of immature myeloid cells (MDSCs, monocytes, tumor associated macrophages) as well as reduced T cell presence, polarization and functionality, as key features of the tumor promotional immune milieu. Consequently many of the immunotherapy agents presently under investigation employ small molecules to block monocyte recruitment, survival or enhance macrophage differentiation pathways [45, 49, 50, 66], or utilize monoclonal antibodies to target inhibitory receptors, or agonize stimulatory receptors on T cells [65]. Unfortunately, immunomodulatory therapies have yet to be broadly successful in breast cancer patients and consequently no immunotherapy agent alone is FDA approved for breast cancer treatment. This is somewhat surprising given the significant correlation between patient survival and gene expression associated with Th1/M1 immune activity that we depicted above (Fig. 8). These observations suggest the need for rationale combination treatments that synergize the activities of both T cells and myeloid cells in order to achieve optimized anti-tumor immunity.

Interestingly, administration of ibuprofen during the window of mammary gland involution leads to changes in abundance and polarization of both of these key immune populations in ways that associate with reduced tumor burden in our postpartum breast cancer model. This observation suggests that a simple, cheap and synergistic benefit to immunotherapy regiments acting in a prostaglandin rich environment, such as mammary gland involution, might be ibuprofen or other NSAID. Recent work validate this approach in rodent preclinical models of melanoma [67, 68], gastric cancer [67], breast cancer [68], and chronic viral exhaustion [69] in which combination treatment of PD-1 with NSAIDs results in dramatically improved T cell responses and tumor outcomes.

Through the utilization of multiplex IHC we were able to make several notable observations regarding T cells in response to the involuting mammary environment with and without administration of ibuprofen. First we were able to observe that involution associated murine mammary tumors possess fewer intratumoral T cells overall, with a specific decrease within the CD4+ T cell compartment of Foxp3+ T cells. Under most circumstances decreasing Foxp3+ T cells would be considered beneficial; however, in this case we hypothesize that the decreased numbers of Foxp3+ T cells is likely to be reflective of the reported ability of PGE2 to desensitize T cells to IL-2 stimulation. Regulatory T cells are particularly sensitive to IL-2 deprivation and consequently, under the influence of PGE2 rich involution environment, may in fact be disproportionally suppressed. NSAID treatment may release this suppression. Despite the evidence that regulatory T cells inhibit anti-tumor responses in the tumor microenvironment and that strategies targeting their depletion in mouse models provide anti-tumor benefit [65, 70], it is interesting to note that in the TCGA breast cancer data set high expression of FOXP3 does not predict poorer outcomes and in fact may correspond with better overall survival (Fig. 8f). One explanation might be that an activated immune system in which all T cell populations rise is a better prognostic indicator than T cell deplete micro-environments.

An additional notable observation from our multiplex IHC examinations included a dramatic decrease in total T cells in the tumor draining lymph node of involution group mice, which is restored with ibuprofen treatment. Furthermore, in the tumor draining lymph node we observed ibuprofen to reduce Rorgt+ CD4+ T cells and restore Granzyme B+ expression in T cells suppressed by the involution environment. These observations indicate that COX-2 inhibition leads to a change in transcriptional programming and activation of cytotoxic T cells, likely consequent of an altered immune milieu. This is consistent with previous reports that have demonstrated that PGE2 amplifies the ability of TGFbeta to enhance Th17 differentiation [29].

At the tumor site itself, ibuprofen treatment increases T cells at the tumor border but does not increase T cell penetration into the tumor. While some aspects of tumor control can be executed at the tumor border, rejection and eradication of established tumors is often associated with tumor infiltrating T cell responses [48, 71]. Thus, lack of T cell infiltration along with the restoration of Treg cell populations with ibuprofen treatment suggests additional benefit, and perhaps synergy, would come from therapies that combine inhibition of COX-2 or PGE2 with therapies targeting regulatory T cell function (i.e. anti-TGFbeta, anti-IL-10) or therapies that enhance T cell trafficking into the tumor (anti-VEGF).

Apart from PGE2 impacting T cells in the tumor microenvironment, the study of PGE2 in myeloid cell biology has a long history. It has been previously reported that PGE2 enhances the presence of myeloid derived suppressor cells, facilitates M2 polarization, enhances mast cell and neutrophil degranulation, and drives production of tolerogenic DCs. Here, we report activities of the COX-2 pathway in the context of involution as broadly impairing macrophage/monocyte differentiation. In our bone marrow progenitor experiments, ibuprofen treatment resulted in a dose dependent increase in expression of the extracellular proteins CD11b and F4/80 indicative of macrophage maturation. Interestingly, in the presence of PGE2, we also see ibuprofen drive specific enhanced gene expression of the cytokines IL-12 and IL-1b consistent with M1 macrophages polarization. These data depict COX-2 inhibition as playing an important role in promoting myeloid cellular differentiation and polarization towards an M1 phenotype when an inflammatory mediator is present, as occurs during involution. Consistent with these in-vitro data, while ibuprofen administration during involution broadly impairs tumor growth to appear more like the nulliparous cohort, the data from Fig. 5 depicts unique tumor immune attributes elicited from the combination of ibuprofen and involution. This illustrates a different “anti-tumor” immune milieu between the nulliparous and the ibuprofen treated involution host, which we suggest is consequent of ibuprofen not simply reversing the massive immune cell recruitment broadly reported in normal involution tissues but instead redirecting the inflammation to an M1/Th1/anti-tumor phenotype. This conclusion is strengthened by our observation that the addition of ibuprofen did not hinder tumor growth in nulliparous hosts where inflammation, immune cell recruitment, and prostaglandin production (COX-2 activity) are relatively minimized (Fig. 9e). Therefore, in the absence of the tumor promotional inflammatory milieu of involution, targeting of tumors that do not overexpress COX-2 (such as the D2A1 mammary tumor cells utilized in these experiments) appears neither helpful nor harmful to tumor outcomes.

While postpartum breast cancer carries a poor prognosis, it is less common than post-menopausal breast cancer, with an estimated ~ 15,000 cases per year in the United States and 160,000 cases worldwide [13]. The population at risk for postpartum breast cancer is equivalent to the number of births per year, with almost 4 million per year in the United States in 2017 (U.S. Census Bureau, Center for Disease Control). Furthermore, our studies as well as others have shown that breast cancer risk from the postpartum period extends out 5–10 years post pregnancy. Consequently, at this very moment a minimum of 20 million women in the United States face poorer survival outcomes if they are diagnosed with breast cancer. From a prevention perspective, given the magnitude of the at-risk population yet relatively low incidence of postpartum breast cancer cases, finding a safe and effective means of interceding in this tumor promotional biology of mammary gland involution through a chemopreventive strategy could be impactful. To date, colon and breast cancer chemopreventive strategies utilizing NSAIDs require chronic use, which can lead to adverse gastric, hepatologic and cardiovascular side effects. Our PPBC studies identify a narrow window of increased COX-2 dependent breast cancer risk and progression, the window of weaning-induced mammary gland involution. Thus, our data suggest NSAID application for PPBC would require only short duration (the months surrounding weaning) and moderate dose use, a strategy anticipated to greatly reduce potential harms associated with long term NSAID use. Through our work with ibuprofen, we show the prostaglandin axis to be a potent immunomodulatory target for the tumor promotional microenvironment of PPBC while remaining a potentially non-deleterious strategy for non-tumor bearing subjects.

## Conclusions

In sum, our data represent an important first step in consideration of moderate dose ibuprofen as a safe intervention in young women during breast involution to protect against the tumor promotional aspects of this unique developmental tissue-remodeling processes. However, we argue that evocation of an altered immune response in the setting of a chemopreventive strategy where tumor presence is unknown requires more careful consideration. Even so, for women diagnosed with postpartum breast cancer, our preclinical data suggest that NSAIDs in combination with immunomodulatory therapy may improve response to treatment, justifying advancement of trials to test this hypothesis.

## Additional files

Additional file 1: **Figure S1.** No alteration in T cell number on the tumor border during involution. Multiplex IHC analysis of the tumor border depicted **a** by hierarchical clustering of all evaluated parameters compared to cohort (black, Nulliparous (NP) or red, Involution (INV)) clustering. Individual parameter analysis of **b** CD45+, CD45 + CD3+, CD45 + CD3 + CD8+, and CD45 + CD3 + CD8- cells in the tumor border region expressed as a percentage of total tumor border nuclei. **c** and fraction of tumor border CD8 T cells positive for granzyme B (Gzm B) and tumor border CD4 T cells positive for Foxp3. No significant differences observed. (TIF 2191 kb)

Additional file 2: **Figure S2.** RNA Seq Analysis of Macrophages and Monocytes. **a** Involution D6 mammary glands were digested into single cell suspension and stained for characterization of F4/80+ populations by flow cytometry. Gates based upon F4/80 expression (orange F4/480-, blue-F4/80+ and red F4/80++) were populated into histogram overlays for comparison of lineage marker expression (CD45, CD11b, Ly6C) and activation (CD70, CD86, CD80) between F4/80+ and F4/80++ groups. **b** Principal component analysis of RNAseq data from involution day 6 mammary gland associated F4/80 low monocytes vs F/480 high macrophages with and without ibuprofen (IBU). **c** Complete gene lists for differentially expressed genes in macrophages (Mac) and monocytes (Mono) with and without ibuprofen treatment. Genes more highly expressed without ibuprofen are in red, while those more highly expressed with ibuprofen are in blue. **d** Examples of GSEA for transcription factor related gene pathways. Analysis by GSEA in which gene sets are composed of genes enriched in response to experimental overexpression of transcription factors (LEF-1) are annotated to have canonical transcription factor binding sites proximal to the indicated gene (STAT5). (TIF 9442 kb)

Additional file 3: **Figure S3.** Induction of COX-2 expression and evaluation of cell death bone marrow derived monocytes. Bone marrow monocyte cultures were established by incubation of bone marrow cells from nulliparous animals in the presence of GM-CSF (20 ng/mL) and IL-4 (10 ng/mL) with or without 100uM concentration of ibuprofen for five days with or without an initial introduction of PGE2 (0.92 ng/mL). **a** Day 5 adherent and non-adherent cells were collected for evaluation of COX-2 protein expression by western blot. **b** MTT assay quantification of viable cells was performed on 24 (gray) and 48 (black) hour ibuprofen treated bone marrow monocyte cultures with increasing concentrations of ibuprofen. Absorbance values for untreated cultures was set as 100% viability. (TIF 2270 kb)

Additional file 4: **Figure S4.** Antigen specific naïve T cell activation schemas. 150,000 Balb/c TCR transgenic CD4+ T cells specific for ovalbumin antigen (DO11.10) were adoptively transferred into Balb/c host that were either nulliparous or had just initiated involution through synchronous weaning (INV D0). Mice either received 300 mg/kg chow ibuprofen or not for the duration of the experiment. Two days post transfer of T cells (INV D2) whole ovalbumin antigen was then introduced locally into the left 4th mammary gland and PBS injected into the contralateral gland. Five days later glands and node were harvested and quantified for antigen specific T cells by flow cytometry for TCR clonotypic antibody staining (KJ1–26) to determine absolute numbers of transgenic T cells. (TIF 1471 kb)
