# Supplementary figures and images for: Ibuprofen supports macrophage differentiation, T cell recruitment, and tumor suppression in a model of postpartum breast cancer
Source: J Immunother Cancer. 2018 Oct 1;6:98. doi: 10.1186/s40425-018-0406-y (PMC6167844; doi:10.1186/s40425-018-0406-y)

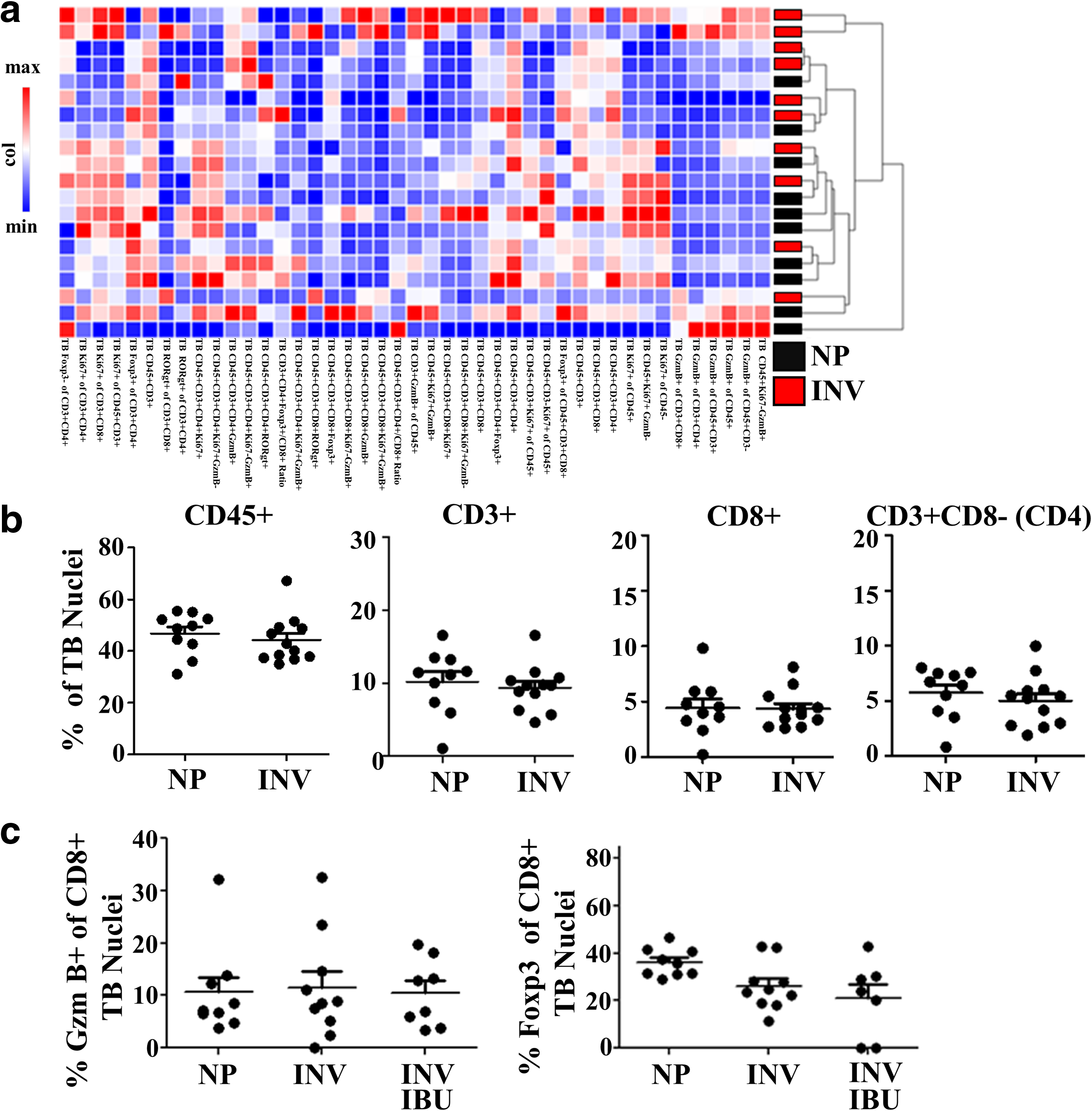

Supplement: Supplementary file 1 — Figure S1. No alteration in T cell number on the tumor border during involution. Multiplex IHC analysis of the tumor border depicted a by hierarchical clustering of all evaluated parameters compared to cohort (black, Nulliparous (NP) or red, Involution (INV)) clustering. Individual parameter analysis of b CD45+, CD45 + CD3+, CD45 + CD3 + CD8+, and CD45 + CD3 + CD8- cells in the tumor border region expressed as a percentage of total tumor border nuclei. c and fraction of tumor border CD8 T cells positive for granzyme B (Gzm B) and tumor border CD4 T cells positive for Foxp3. No significant differences observed. (TIF 2191 kb) [file 40425_2018_406_MOESM1_ESM.tif]

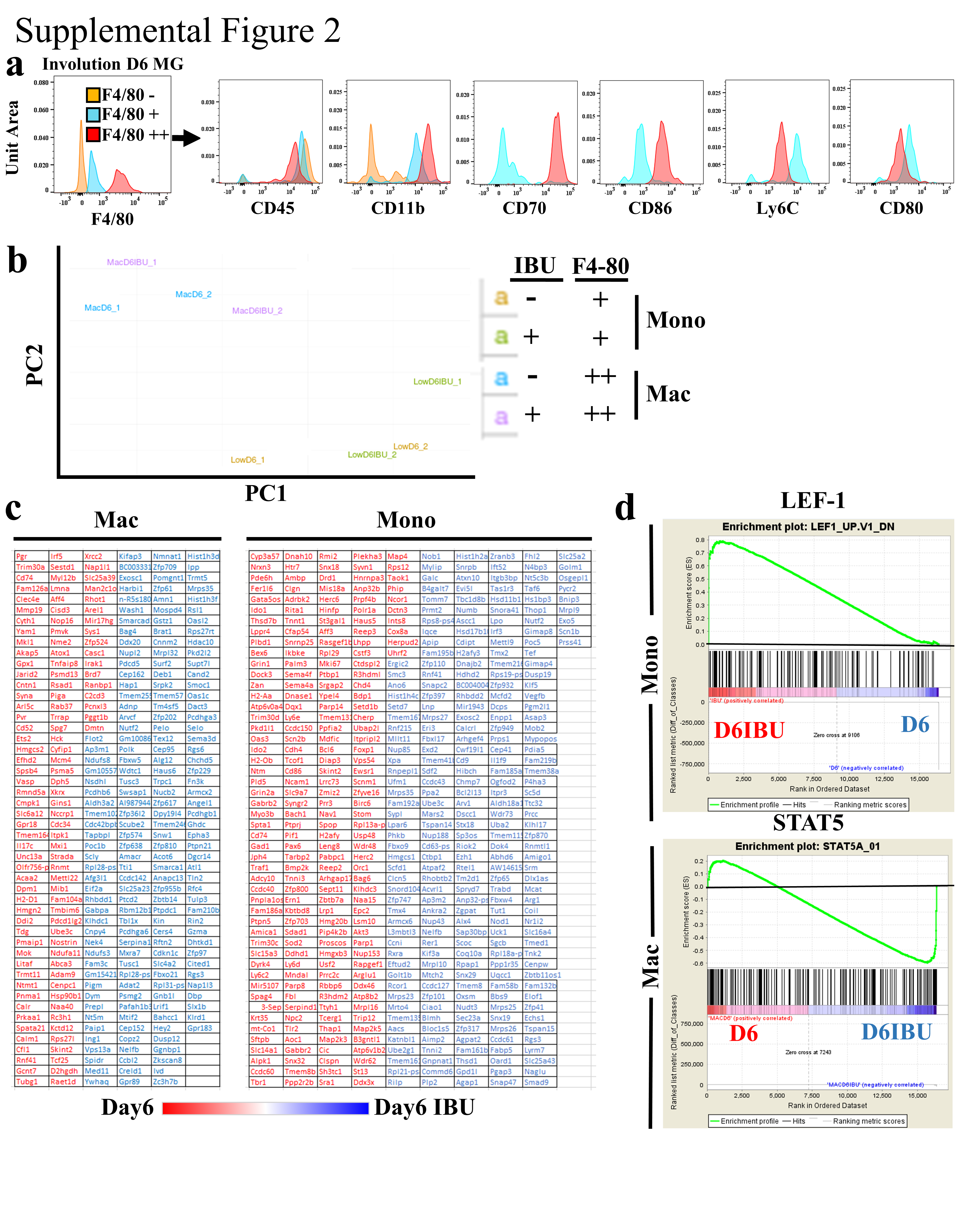

Supplement: Supplementary file 2 — Figure S2. RNA Seq Analysis of Macrophages and Monocytes. a Involution D6 mammary glands were digested into single cell suspension and stained for characterization of F4/80+ populations by flow cytometry. Gates based upon F4/80 expression (orange F4/480-, blue-F4/80+ and red F4/80++) were populated into histogram overlays for comparison of lineage marker expression (CD45, CD11b, Ly6C) and activation (CD70, CD86, CD80) between F4/80+ and F4/80++ groups. b Principal component analysis of RNAseq data from involution day 6 mammary gland associated F4/80 low monocytes vs F/480 high macrophages with and without ibuprofen (IBU). c Complete gene lists for differentially expressed genes in macrophages (Mac) and monocytes (Mono) with and without ibuprofen treatment. Genes more highly expressed without ibuprofen are in red, while those more highly expressed with ibuprofen are in blue. d Examples of GSEA for transcription factor related gene pathways. Analysis by GSEA in which gene sets are composed of genes enriched in response to experimental overexpression of transcription factors (LEF-1) are annotated to have canonical transcription factor binding sites proximal to the indicated gene (STAT5). (TIF 9442 kb) [file 40425_2018_406_MOESM2_ESM.tif]

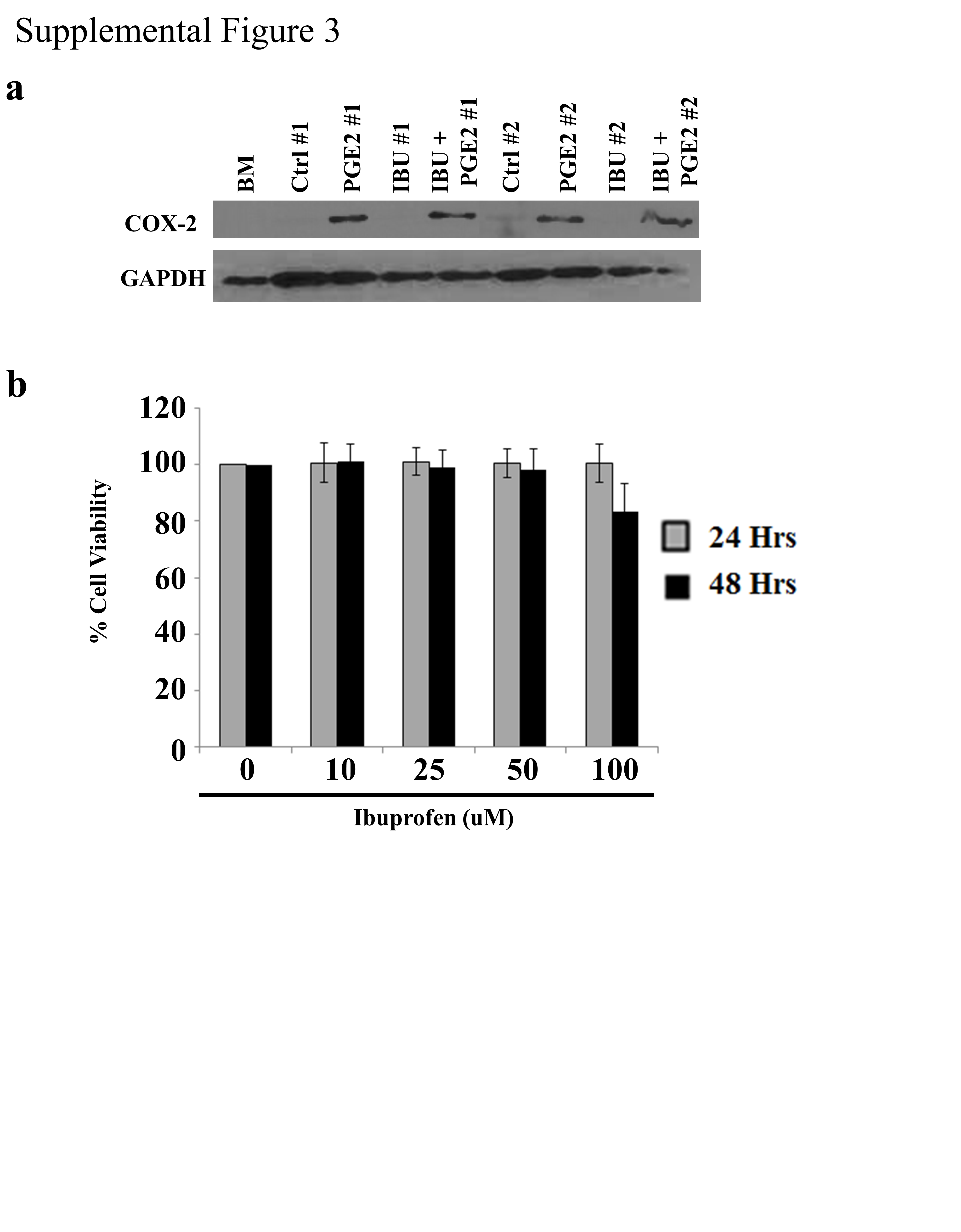

Supplement: Supplementary file 3 — Figure S3. Induction of COX-2 expression and evaluation of cell death bone marrow derived monocytes. Bone marrow monocyte cultures were established by incubation of bone marrow cells from nulliparous animals in the presence of GM-CSF (20 ng/mL) and IL-4 (10 ng/mL) with or without 100uM concentration of ibuprofen for five days with or without an initial introduction of PGE2 (0.92 ng/mL). a Day 5 adherent and non-adherent cells were collected for evaluation of COX-2 protein expression by western blot. b MTT assay quantification of viable cells was performed on 24 (gray) and 48 (black) hour ibuprofen treated bone marrow monocyte cultures with increasing concentrations of ibuprofen. Absorbance values for untreated cultures was set as 100% viability. (TIF 2270 kb) [file 40425_2018_406_MOESM3_ESM.tif]

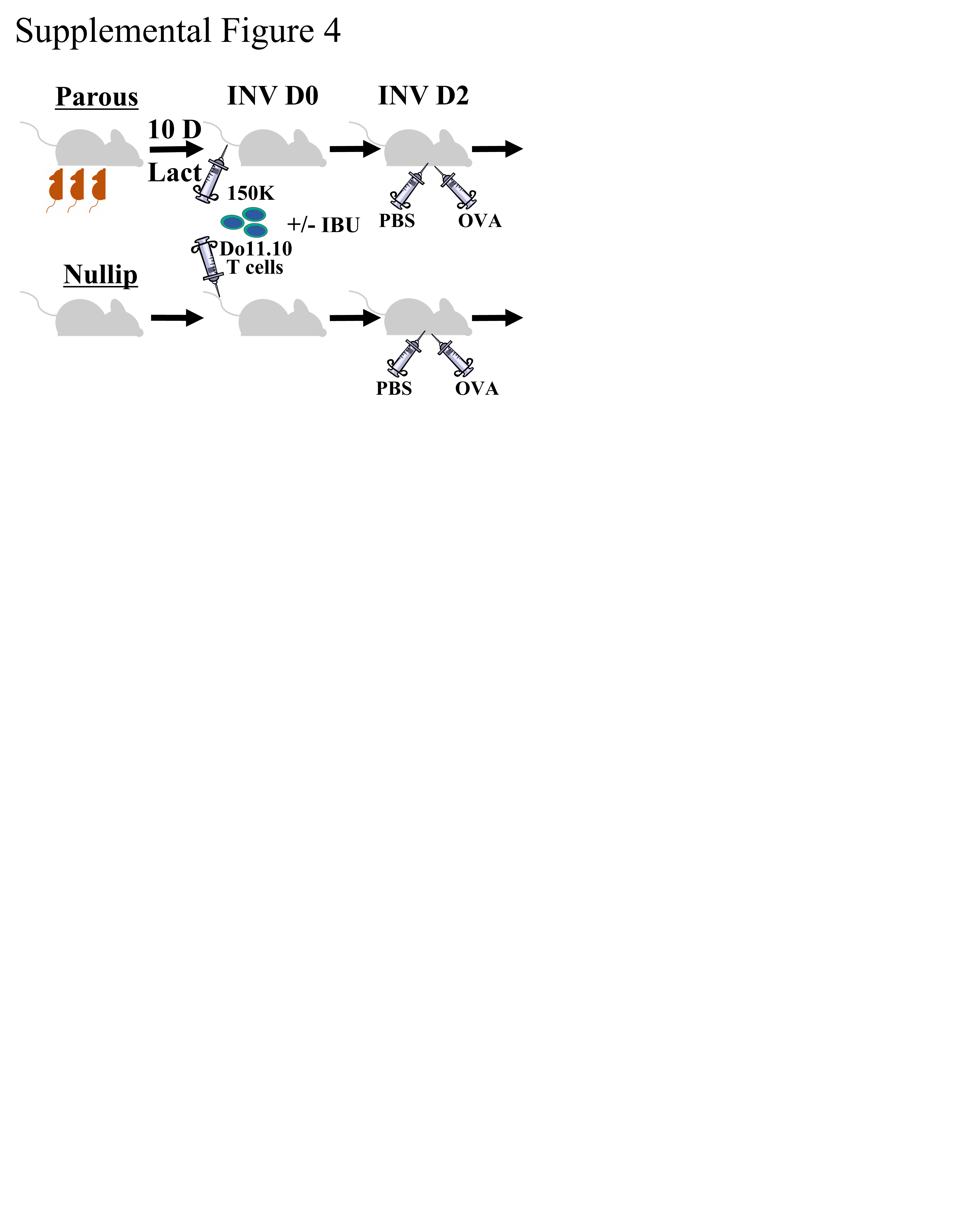

Supplement: Supplementary file 4 — Figure S4. Antigen specific naïve T cell activation schemas. 150,000 Balb/c TCR transgenic CD4+ T cells specific for ovalbumin antigen (DO11.10) were adoptively transferred into Balb/c host that were either nulliparous or had just initiated involution through synchronous weaning (INV D0). Mice either received 300 mg/kg chow ibuprofen or not for the duration of the experiment. Two days post transfer of T cells (INV D2) whole ovalbumin antigen was then introduced locally into the left 4th mammary gland and PBS injected into the contralateral gland. Five days later glands and node were harvested and quantified for antigen specific T cells by flow cytometry for TCR clonotypic antibody staining (KJ1–26) to determine absolute numbers of transgenic T cells. (TIF 1471 kb) [file 40425_2018_406_MOESM4_ESM.tif]
